# Migratory hosts can maintain the high‐dose/refuge effect in a structured host‐parasite system: The case of sea lice and salmon

**DOI:** 10.1111/eva.12984

**Published:** 2020-09-10

**Authors:** Andrew W. Bateman, Stephanie J. Peacock, Martin Krkošek, Mark A. Lewis

**Affiliations:** ^1^ Pacific Salmon Foundation Vancouver BC Canada; ^2^ Salmon Coast Field Station Simoom Sound BC Canada; ^3^ Department of Biological Sciences University of Calgary Calgary AB Canada; ^4^ Department of Ecology and Evolutionary Biology University of Toronto Toronto ON Canada; ^5^ Department of Biological Sciences University of Alberta Edmonton AB Canada; ^6^ Department of Mathematical and Statistical Sciences University of Alberta Edmonton AB Canada

**Keywords:** emamectin benzoate, high dose/refuge effect, host–parasite model, salmon, salmon farming, sea lice, treatment resistance

## Abstract

Migration can reduce parasite burdens in migratory hosts, but it connects populations and can drive disease dynamics in domestic species. Farmed salmon are infested by sea louse parasites, often carried by migratory wild salmonids, resulting in a costly problem for industry and risk to wild populations when farms amplify louse numbers. Chemical treatment can control lice, but resistance has evolved in many salmon‐farming regions. Resistance has, however, been slow to evolve in the north‐east Pacific Ocean, where large wild‐salmon populations harbour large sea louse populations. Using a mathematical model of host–macroparasite dynamics, we explored the roles of domestic, wild oceanic and connective migratory host populations in maintaining treatment susceptibility in associated sea lice. Our results show that a large wild salmon population, unexposed to direct infestation by lice from farms; high levels of on‐farm treatment; and a healthy migratory host population are all critical to slowing or stopping the evolution of treatment resistance. Our results reproduce the “high‐dose/refuge effect,” from the agricultural literature, with the added requirement of a migratory host population to maintain treatment susceptibility. This work highlights the role that migratory hosts may play in shared wildlife/livestock disease, where evolution can occur in ecological time.

## 1 INTRODUCTION

Long‐distance migration is a striking feature of many animals' life histories, and one that is closely tied to disease. Migration can offer refuge from sources of infection, and reduced parasite loads in some migrants suggest the role parasites may have in the evolution of migration itself (Bartel, Oberhauser, De Roode, & Altizer, 2011; Folstad, Nilssen, Halvorsen, & Andersen, 1991; Mijele et al., 2016). Migration can, however, transfer pathogenic organisms between hosts in different environments. Migrating wildebeests (*Connochaetes taurinus*), for example, display lower helminth parasite diversity than their sessile counterparts, but the parasites they do harbour are generalists that also infest livestock, emphasizing the risk of cross‐infection in such scenarios (Mijele et al., 2016). Migration can even drive disease dynamics, as in the case of wild‐bird migrations that lead to periodic re‐emergence of H5N1 avian influenza in Asia (Tian et al., 2015). In general, disease transmission between wildlife and livestock is an area of concern for human health, agriculture and wildlife conservation (Daszak, Cunningham, & Hyatt, 2000). Migration can also have complex impacts on evolutionary processes that affect the ability to manage disease (e.g. Bonnedahl & Järhult, 2014; Comins, 1977). Here, we use a model to highlight the beneficial role, for both a wild and a farmed species, that migration and related cross‐infection might play in maintaining the efficacy of disease management by reducing the build‐up of resistance to chemical therapeutants designed to control the disease.

The use of chemicals to protect against pathogens is vital to human, livestock and crop health, but treatment presents its own problems, one of which is that it selects for the evolution of treatment resistance in target organisms (Aaen, Helgesen, Bakke, Kaur, & Horsberg, 2015; Austin, Kristinsson, & Anderson, 1999; Comins, 1977). Management of treatment resistance has been well studied in the cases of antibiotic resistance in pathogenic bacteria (Neu, 1992; Normark & Normark, 2002) and insecticide resistance in insect pests of crop plants (Comins, 1977; Tabashnik, Brévault, & Carrière, 2013). Paradoxically, while the frequency of antibiotic resistance in bacteria increases with the rate of antibiotic administration (Austin et al., 1999), high treatment levels combined with the provision of a treatment‐free refuge have been suggested as a means to slow or preclude the emergence of treatment‐resistant crop pests (Comins, 1977). When resistance is sufficiently recessive, susceptible migrants from the untreated refuge can mate with pests in the treated habitat, producing susceptible heterozygotes and facilitating the purging of resistance genes by further treatment (Comins, 1977). In the case of crops genetically engineered to express *Bacillus thuringiensis* (Bt) toxin to deter pests, taking advantage of such a “high‐dose/refuge” (HDR) effect has now entered into standard practice (Gould, 1998; Tabashnik et al., 2013). Critically, the HDR effect relies on the ability of pests or pathogens to migrate between the treated and untreated environments.

Recent domestication of animals for aquaculture has occurred at an unprecedented rate (Duarte, Marbá, & Holmer, 2007), leading to growing stocks of domesticated fish and shellfish (FAO, 2016) and increasing potential for the emergence of diseases shared by wild and domesticated hosts (Daszak et al., 2000). Pathogens, including parasites, associated with aquaculture can imperil populations of wild species (Daszak et al., 2000; Krkošek, 2017) and carry substantial costs for aquaculture operations (Lafferty et al., 2015), where pesticide and antibiotic treatments are commonly used as control measures. Resultant treatment resistance can threaten both aquaculture operations' profitability and their capacity to guard wild populations against disease.

In Atlantic salmon (*Salmo salar*) aquaculture, one group of ectoparasites—sea lice (*Lepeophtheirus salmonis* and *Caligus* spp.)—presents particular problems. Sea lice cost the global salmon‐farming industry several 100 M USD annually (Abolofia, Asche, & Wilen, 2017; Costello, 2009), and sea lice from salmon farms have been linked to nearby wild salmon declines (Ford & Myers, 2008; Krkošek, Connors, Morton, et al., 2011; Vollset et al., 2016), although the magnitude of impacts is debated (e.g. Torrissen et al., 2013). In coastal British Columbia (BC), some authors claim that populations of wild pink salmon (*Oncorhynchus gorbuscha*) have recovered from sea lice‐associated population collapse in the early 2000s due to effective treatment with emamectin benzoate (EMB, industry name: SLICE) on salmon farms (Peacock, Krkosek, Proboszcz, Orr, & Lewis, 2013, but see Brooks and Jones, 2008). Resistance to EMB and other drugs has evolved globally over the past decades (Aaen et al., 2015), but resistance to EMB has been much slower to evolve in BC sea lice and has tended to be ephemeral or localized when it has arisen (Bateman et al., 2016; Messmer et al., 2018; Saksida et al., 2013). Nonetheless, recent localized emergence of resistance in BC has alarmed observers (e.g. Thomas, 2018, popular press article). Understanding and managing the factors that may affect resistance evolution in this system is therefore important for salmon farmers, wild salmon and those that depend on wild salmon.

*Lepeophtheirus salmonis*, often called the salmon louse, is an obligate salmon parasite. Hatched into the water column as free‐swimming nauplius larvae, salmon lice moult into infective copepodid larvae that can attach to salmon hosts and then moult through tethered chalimus and motile pre‐adult stages, before moulting into sexually reproductive adults (Hamre, Eichner, & Caipang, 2013). Maturation and development are highly temperature‐dependent, but females, maturing one to two months after initial attachment, can release several hundred larvae every week or two (Heuch, Nordhagen, & Schram, 2000). Salmon farms, commonly sited in sheltered coastal waters, break the migratory allopatry that separates juvenile pink salmon from adults and protects the juveniles from adult‐origin sea louse infestation (Krkošek et al., 2007). Farms provide a novel, high‐density source of hosts for sea lice, creating the potential for outbreaks that infest wild juvenile salmon (Frazer, Morton, & Krkošek, 2012).

Past mathematical and simulation‐based models have explored the interplay between antiparasite treatment and host abundance in the context of sea lice and salmon. One of the first studies (Murray, 2011) showed that moderate treatment rates and high wild/farm louse transfer rates could lead to higher levels of treatment resistance. A later simulation‐based study showed that larger wild‐host “refugia” and higher fitness costs associated with treatment resistance could substantially slow the rate of resistance evolution (McEwan, Groner, Fast, Gettinby, & Revie, 2015). These modelling efforts, however, envisioned scenarios most similar to those seen in the Atlantic Ocean, where farmed salmon outnumber wild salmon, and treatment resistance is common (Kreitzman et al., 2017). Work focused on the Pacific Ocean has confirmed that connectivity between farm environments and a large wild‐salmon refuge population could slow—or even stop—the evolution of treatment resistance in lice (Ashander, 2010; Kreitzman et al., 2017). It also suggests that the health of wild‐salmon populations plays an important role in the rate of resistance evolution, because individuals spawn near farms but spend a portion of their lives at sea, thereby connecting treated and untreated host populations (Ashander, 2010; Kreitzman et al., 2017). If salmon farms do jeopardize the health of those wild‐salmon populations (e.g. Krkošek, Connors, Morton, et al., 2011), EMB treatment susceptibility of lice on farms could be compromised.

Mechanisms of EMB resistance in sea lice are not known definitively, but insight has been gained from genetic and genomic studies. Multiple putative candidate resistance genes have been identified (Besnier et al., 2014; Igboeli, Fast, Heumann, & Burka, 2012; Messmer et al., 2018; Poley, Igboeli, & Fast, 2015), and there is evidence that resistance is polygenic, at least in Atlantic sea lice (Espedal, Glover, Horsberg, & Nilsen, 2013; Poley et al., 2015; Sutherland et al., 2015). While there is no guarantee that resistance should involve the same genetic changes if it evolves independently in multiple locations, resistance and reduced sensitivity to EMB have been traced to linkage group five, within the sea louse genome, in both Atlantic (Besnier et al., 2014) and Pacific (Messmer et al., 2018) sea lice. Furthermore, the presence of a widespread resistant haplotype in the Atlantic (Besnier et al., 2014) and strong genetic clustering of reduced‐sensitivity lice in the Pacific (Messmer et al., 2018) suggest a single emergence in each case, followed by spread within respective sea louse populations.

The nature of treatment, and associated strength of selection, is likely to influence the genetic basis of resistance (single‐gene versus polygenic) that evolves as a result (ffrench‐Constant, Daborn, & Le Goff, 2004). If some lice were to receive a sublethal treatment dose, such that selection occurred within the “normal range” of EMB tolerance, resultant resistance would likely be polygenic (ffrench‐Constant et al., 2004; Sutherland et al., 2015). If treatment were to cause near‐complete mortality, however, resistance would more likely result from rare mutations of large effect within single genes (ffrench‐Constant et al., 2004). Such considerations are relevant in formulating and interpreting models of resistance evolution.

To explore the potential for a high‐dose/refuge (HDR) strategy to slow or stop the emergence of EMB resistance in sea lice on salmon farms, we develop and analyse a mathematical model of host–parasite dynamics that includes features of the wild/farmed salmon system relevant to the evolution of treatment resistance. Expanding on the work of Murray (Murray, 2011) and McEwan (McEwan et al., 2015), we explicitly include a population of oceanic salmon, with associated sea lice, that acts as a treatment‐free refuge, as occurs for Pacific salmon. Unlike previous models focused on the Pacific (Ashander, 2010), we use an implicit model of clonal genetics, focusing on the ecological influences on discrete treatment‐susceptible and treatment‐resistant louse morphs after a single emergence of resistance. We show that resistance emergence in this clonal model aligns most closely with that predicted assuming a single‐gene basis of resistance, which we might expect to result from a high‐treatment‐mortality scenario associated with an HDR strategy. We consider the influence of treatment rates, the size of the treatment‐free wild‐salmon refuge, the dynamics of the connective wild‐salmon population that spawns near farms, parasite transfer rates and the parasite fitness costs of resistance. Our theoretical results are relevant for understanding the potential, or lack thereof, for evolution in ecological time of treatment resistance in sea lice associated with salmon farms located in different salmon‐farming regions.

## 2 MODEL

**Figure 1:**
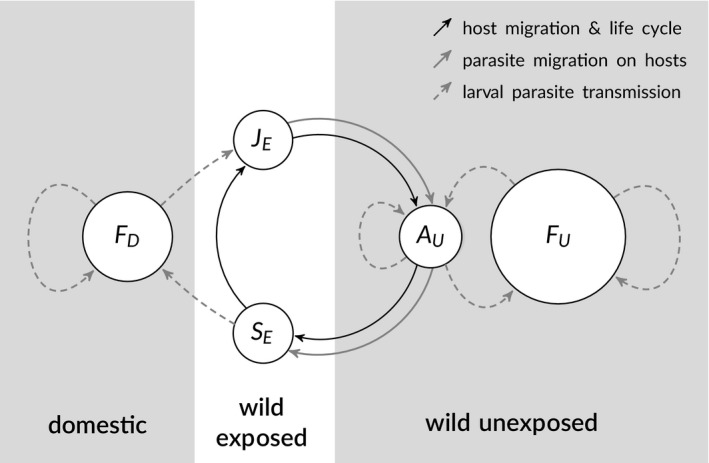
Movement of salmon and sea lice in a differential‐equation model of treatment resistance in sea lice on salmon farms. The model describes domestic fish hosts (*F_D_*); hosts unexposed to direct farm influences (*F_U_*); and a migratory population of hosts that are exposed to farms as juveniles (*J_E_*), migrate to the unexposed environment where they mature into adults (*A_U_*) and return to the exposed environment as spawners (*S_E_*). Treatment‐susceptible and treatment‐resistant sea lice form reproductive populations on hosts in the domestic and unexposed environments, and exposed hosts carry lice between those two environments. See text for details

Our model is a modification of a standard host–macroparasite population model (Anderson & May, 1978), similar to others used in the past to study the salmon–sea lice system (Krkošek, Connors, Ford, et al., 2011; Murray, 2011; Peacock, Connors, Krkosek, Irvine, & Lewis, 2014), but it includes new features to explore the eco‐evolutionary dynamics of the system. The model includes treatment‐resistant and treatment‐susceptible sea louse parasites, fixed domestic (i.e. farmed) and wild salmon host populations, and a dynamic wild host population that migrates between environments near each of the other two host populations (the key addition in our model). The domestic hosts constitute a treated environment for the sea louse parasites, within which selection favours resistance to treatment, while the unexposed, oceanic wild‐host environment constitutes an untreated refuge from selection for resistance. Wild hosts migrating past salmon farms serve as the vector that connects louse populations in the treated and untreated environments (Figure 1). Details of the model derivation are in the Appendix, but we provide a brief summary and the final model below. Variables and parameters are summarized in Table 1.

**Table 1:** Variables and parameters

Symbol	Interpretation	Units
*F_X_*	Host fish (salmon) population in environment *X* (D = domestic; U = unexposed wild, oceanic)	salmon
*J_E_*	Juvenile host fish in the exposed wild environment	salmon
*A_U_*	Adult host fish that mature from juveniles in the exposed environment as they migrate to the unexposed environment	salmon
*S_E_*	Spawning host fish in the exposed wild environment	salmon
*L_sX_* (*L_rX_*)	Susceptible (resistant) adult parasitic sea louse population *y* (*r* = resistant, *s* = Susceptible) in environment *X*	lice
*C_sX_* (*C_rX_*)	Resistant (susceptible) infestive copepodid population in environment *X*	larvae
*t*	Time	years
$\beta_{0,X_{2}X_{1}}$	Transmission rate for infestive parasite larvae produced by adults on hosts in environment *X*_1_ infesting hosts in environment *X*_2_	salmon^−1^year^−1^
ψ	Probability with which attached copepodid lice survive and mature into adult lice	lice∙larva^−1^
$\beta_{X_{2}X_{1}}$	Transmission and maturation rate ( $\beta_{0,X_{2}X_{1}}\cdot \psi$) for parasite larvae produced in environment *X*_1_ infesting hosts in environment *X*_2_	salmon^−1^year^−1^
*µ_s_* (*µ_r_*)	Background mortality rate for susceptible (resistant) lice	year^−1^
*µ_J_*	Background mortality rate juvenile hosts	year^−1^
*γ_T_*	Treatment intensity, modelled as the increase in louse mortality for each additional louse per host	salmon∙lice^−1^year^−1^
*ε_r_*	Effectiveness of treatment for resistant lice	year^−1^
*g*	Farm stocking rate	year^−1^
*h*	Farm harvest rate	year^−1^
*a_0_*	Maximum growth rate of juvenile exposed hosts, not accounting for mortality	salmon∙year^−1^
*b*	Juvenile exposed‐host population size at which growth rate of those juveniles reaches half its maximum ( $b_{0}=m_{\text{out}}b/\sigma$ is the analogue for spawner population size)	salmon
*m*_out_ (*m*_in_)	Migration rate of host fish, to (from) the open ocean	year^−1^
σ	“Spawn‐out” rate for adult fish after migrating back to the nearshore environment	year^−1^
α	Strength of linear parasite‐induced host mortality for juvenile hosts	salmon∙louse^−1^year^−1^
δ	Strength of density‐dependent parasite mortality on wild adults	salmon∙louse^−1^year^−1^
$\lambda_{s}\left(\lambda_{r}\right)$	Production rate for infectious‐stage susceptible (resistant) parasites	larvae∙louse^−1^year^−1^
*c*	Mortality rate of infectious‐stage parasites	year^−1^

The final model tracks susceptible and resistant adult lice, *L_s_* and *L_r_*, respectively, that disperse via larvae released into the water column. These larvae go on to infest salmon in nearby environments. Larvae produced on farms re‐infest domestic hosts and also infest exposed‐juvenile wild hosts as they swim past. As an example of larval louse dynamics in our model, the abundance of infestive larval lice, originating from resistant sea lice on salmon farms, changes according to:

(1) $$\frac{dC_{\mathrm{rD}}}{\mathrm{dt}}=\underset{\text{Larval}\;\text{release}}{\underbrace{\lambda_{r}L_{\mathrm{rD}}}}\overset{\text{Mortality}}{\overbrace{-cC_{\mathrm{rD}}}}\underset{\text{Attachment}\;\;\text{to}\;\;\text{exposed}\;\text{juvenile}\;\text{hosts}}{\underbrace{-\beta_{0,ED}C_{\mathrm{rD}}J_{E}}}\overset{\text{Attachment}\;\text{to}\;\text{domestic}\;\text{hosts}}{\overbrace{-\beta_{0,DD}C_{\mathrm{rD}}F_{D}}}$$

We make standard assumptions (May & Anderson, 1979) that larval parasite mortality is much greater than attachment rates and that larval abundance equilibrates quickly, relative to other model dynamics, so that $C_{\mathrm{rD}}=\lambda_{r}L_{\mathrm{rD}}/c$. Following the larvae after they attach to exposed wild hosts, the dynamics of the attached adult louse population proceeds according to:

(2) $$\frac{dL_{\mathrm{rE}}}{\mathrm{dt}}=\overset{\text{Larval}\;\text{attachment}\;\text{and}\;\text{maturation}}{\overbrace{\psi \beta_{0,ED}C_{\mathrm{rD}}J_{E}}}\underset{\text{Migration},\;\text{natural}\;\text{mortality},\;\text{and}\;\text{mortality}\;\text{due}\;\text{to}\;\text{natural}\;\text{host}\;\text{mortality}}{\underbrace{-\left(m_{\mathrm{out}}+\mu_{r}+\mu_{J}\right)L_{\mathrm{rE}}}}\overset{\text{Mortality}\;\text{due}\;\text{to}\;\text{parasite}-\text{induced}\;\text{host}\;\text{mortality}}{\overbrace{-\alpha L_{\mathrm{rE}}\left[1+\left(\frac{L_{\mathrm{rE}}+L_{\mathrm{sE}}}{J_{E}}\right)\right]}}$$

The final nonlinear term, describing louse mortality due to parasite‐induced host mortality, arises because we assume that each additional louse on a host increases the rate at which that host dies (Appendix A.1). To facilitate consideration of both resistant and susceptible lice on the same hosts, we make the simplifying assumption that the two types of lice are independently Poisson‐distributed (Appendix A.1), as opposed the more general assumption of negative‐binomially distributed lice (May & Anderson, 1979). While assuming a negative binomial distribution for lice would be more general, it would also imply an associated clustering process for each louse morph. We thought it unreasonable to assume that such a clustering process would occur for each type of louse independently, and we decided that accounting for a full dependent joint distribution of susceptible and resistant lice was beyond the scope of this analysis. We consider the implications of this assumption on our conclusions in the Discussion.

We treat resistant and susceptible lice as clonal morphs, ignoring explicit details of genetics. We show in Appendix A.5, however, that the invasion properties of our model match those of a model in which we assume that resistance arises due to a mutant allele of a single gene.

The juvenile exposed hosts, *J_E_*, carry lice to the oceanic, unexposed environment. There, those juvenile hosts mature into adults, *A_U_*, and mingle with other wild adult hosts, *F_U_*, that originated away from salmon farms. Lice in the unexposed environment persist and re‐infest the unexposed hosts but cannot infest nearshore domestic hosts. After maturing in the unexposed environment, hosts originally from the exposed environment migrate back to coastal waters, becoming spawners, *S_E_*, and carrying lice with them as they pass by the domestic hosts, *F_D_*. At that point, larval lice produced by adult lice on the returning spawners can infest domestic hosts. Note, however, that these lice do not infest juvenile wild hosts in the exposed environment, as we are using the model to represent a system in which migratory allopatry separates juvenile salmon from returning adults (Krkošek et al., 2007).

For hosts that spawn in the exposed environment, the life cycle becomes:

(3a) $$\frac{dJ_{E}}{\mathrm{dt}}=\overset{\text{Density}-\text{dependent}\;\text{reproduction}}{\overbrace{\frac{a_{0}S_{E}}{b_{0}+S_{E}}}}\underset{\text{Migration}\;\text{and}\;\text{natural}\;\text{mortality}}{\underbrace{-\left(m_{\text{out}}+\mu_{J}\right)J_{E}}}\overset{\text{Parasite}-\text{induced}\;\text{mortality}}{\overbrace{-\alpha \left(L_{\mathrm{sE}}+L_{\mathrm{rE}}\right)}}$$

(3b) $$\frac{dA_{U}}{\mathrm{dt}}=m_{\text{out}}J_{E}-m_{\text{in}}A_{U}$$

(3c) $$\frac{dS_{E}}{\mathrm{dt}}=m_{\text{in}}A_{U}-\sigma S_{E}$$

For pink salmon, which we use as a case study, wild‐host population dynamics occur on a fixed two‐year generation time. We assume that exposed‐host dynamics are much faster than the resistance‐frequency dynamics with which we are concerned, and we therefore make the quasi‐steady‐state assumption that exposed adults and spawners are at equilibrium for a given population of juveniles. As a result, in our final model, we only explicitly track exposed juveniles and not the corresponding adults or spawners. That is, we assume pink salmon population dynamics stabilize faster than lice evolve, and we simplify the model to describe the dynamics of the migratory population based solely on its juvenile abundance.

As pink salmon age, they develop scales and immune defences that better protect them from the effects of sea lice (Jones, Kim, & Bennett, 2008; Krkošek et al., 2007), making parasite‐induced mortality much less likely. We therefore assume that only juvenile wild salmon experience parasite‐induced host mortality and that unexposed‐host abundance is fixed. Given that sea louse numbers on wild salmon in the Pacific Ocean appear, empirically, to be bounded (Gottesfeld, Proctor, Rolston, & Carr‐Harris, 2009), we also assume that louse abundance in the unexposed environment is regulated by density‐dependent louse mortality (at rate δ per unexposed louse on each host in the population). Farmed salmon are added to ocean net pens at a more mature stage than that at which sympatric wild pink salmon enter the ocean (Groot & Margolis, 1991), and farmed salmon are then managed carefully for parasites (Abolofia et al., 2017). Thus, while sea lice may affect the profitability of farmed salmon, we assume that their effect on farmed salmon mortality is negligible. We implicitly assume that a constant farm stocking rate balances constant mortality and harvest rates, within our modelled salmon‐farming region as a whole, and so farm‐host abundance is fixed in our model. As a result, lice on domestic hosts are not regulated by parasite‐induced host mortality but by direct treatment mortality, with the mortality rate increasing by γ_T_ for each domestic louse per host for susceptible lice and by *ε_r_γ_T_* for each domestic louse per host (where ε_r_ < 1) for resistant lice. The *γ_T_* parameter represents treatment intensity, which would normally be altered by changes in treatment frequency, but which enters our continuous‐time model as a rate parameter.

In the full model, we track six populations of lice (resistant and susceptible lice in farm, exposed and unexposed environments), as sketched in Equation 2. We make two quasi‐steady‐state substitutions (Appendix A.2): (1) that adult hosts originating in the exposed environment, *A_U_*, are equal to $\left(m_{\text{out}}/m_{\text{in}}\right)J_{E}$, and (2) that spawners returning to the exposed environment, *S_E_*, are equal to $\left(m_{\text{out}}/\sigma \right)J_{E}$. The first approximation is relevant for louse dynamics in the unexposed environment and the second for connecting the louse population in the unexposed and domestic environments. The full model becomes:

(4a) $$\frac{dF_{D}}{\mathrm{dt}}=0$$

(4b) $$\frac{dL_{\mathrm{sD}}}{\mathrm{dt}}=\frac{\lambda_{s}}{c}\left[\beta_{\mathrm{DD}}L_{\mathrm{sD}}+\beta_{\mathrm{DE}}L_{\mathrm{sU}}\frac{\frac{m_{\text{out}}}{\sigma }J_{E}}{\left(F_{U}+\frac{m_{\mathrm{out}}}{m_{\mathrm{in}}}J_{E}\right)}\right]F_{D}-\left(\mu_{s}+h\right)L_{\mathrm{sD}}-\gamma_{T}\left(\frac{L_{\mathrm{rD}}+L_{\mathrm{sD}}}{F_{D}}\right)L_{\mathrm{sD}}$$

(4c) $$\frac{dL_{\mathrm{rD}}}{\mathrm{dt}}=\frac{\lambda_{r}}{c}\left[\beta_{\mathrm{DD}}L_{\mathrm{rD}}+\beta_{\mathrm{DE}}L_{\mathrm{rU}}\frac{\frac{m_{\text{out}}}{\sigma }J_{E}}{\left(F_{U}+\frac{m_{\text{out}}}{m_{\text{in}}}J_{E}\right)}\right]F_{D}-\left(\mu_{r}+h\right)L_{\mathrm{rD}}-\varepsilon_{r}\gamma_{T}\left(\frac{L_{\mathrm{rD}}+L_{\mathrm{sD}}}{F_{D}}\right)L_{\mathrm{rD}}$$

(4d) $$\frac{dJ_{E}}{\mathrm{dt}}=\frac{a_{0}J_{E}}{b+J_{E}}-\left(m_{\text{out}}+\mu_{J}\right)J_{E}-\alpha \left(L_{\mathrm{sE}}+L_{\mathrm{rE}}\right)$$

(4e) $$\frac{dL_{\mathrm{sE}}}{\mathrm{dt}}=\frac{\lambda_{s}}{c}\beta_{\mathrm{ED}}L_{\mathrm{sD}}J_{E}-\left(m_{\text{out}}+\mu_{s}+\mu_{J}+\alpha \right)L_{\mathrm{sE}}-\alpha \left(\frac{L_{\mathrm{rE}}+L_{\mathrm{sE}}}{J_{E}}\right)L_{\mathrm{sE}}$$

(4f) $$\frac{dL_{\mathrm{rE}}}{\mathrm{dt}}=\frac{\lambda_{r}}{c}\beta_{\mathrm{ED}}L_{\mathrm{rD}}J_{E}-\left(m_{\text{out}}+\mu_{r}+\mu_{J}+\alpha \right)L_{\mathrm{rE}}-\alpha \left(\frac{L_{\mathrm{rE}}+L_{\mathrm{sE}}}{J_{E}}\right)L_{\mathrm{rE}}$$

(4g) $$\frac{dF_{U}}{\mathrm{dt}}=0$$

(4h) $$\frac{dL_{\mathrm{sU}}}{\mathrm{dt}}=\frac{\lambda_{s}}{c}\beta_{\mathrm{UU}}L_{\mathrm{sU}}\left(F_{U}+\frac{m_{\text{out}}}{m_{\text{in}}}J_{E}\right)+m_{\text{out}}L_{\mathrm{sE}}-\left(m_{\text{in}}+\mu_{s}+\delta \right)L_{\mathrm{sU}}-\delta \frac{L_{\mathrm{rU}}+L_{\mathrm{sU}}}{\left(F_{U}+\frac{m_{\text{out}}}{m_{\text{in}}}J_{E}\right)}L_{\mathrm{sU}}$$

(4i) $$\frac{dL_{\mathrm{rU}}}{\mathrm{dt}}=\frac{\lambda_{r}}{c}\beta_{\mathrm{UU}}L_{\mathrm{rU}}\left(F_{U}+\frac{m_{\text{out}}}{m_{\text{in}}}J_{E}\right)+m_{\text{out}}L_{\mathrm{rE}}-\left(m_{\text{in}}+\mu_{r}+\delta \right)L_{\mathrm{rU}}-\delta \frac{L_{\mathrm{rU}}+L_{\mathrm{sU}}}{\left(F_{U}+\frac{m_{\text{out}}}{m_{\text{in}}}J_{E}\right)}L_{\mathrm{rU}}$$

### 2.1 Model analysis

#### 2.1.1 Net reproductive number, *R*_0_

In the Appendix, we detail calculation of the net reproductive number, *R*_0_, for lice in our model, as well as farm‐only and wild‐only submodels. For resistant lice invading the overall system, assuming hosts and susceptible lice are at equilibrium:

(5) $$R_{0,r}=\frac{1}{2}\left(\frac{f_{r,11}}{v_{r,11}}+\frac{f_{r,33}}{v_{r,33}}\right)+\sqrt{{\left[\frac{1}{2}\left(\frac{f_{r,11}}{v_{r,11}}+\frac{f_{r,33}}{v_{r,33}}\right)\right]}^{2}-\left(\frac{f_{r,11}}{v_{r,11}}\frac{f_{r,33}}{v_{r,33}}+\frac{f_{r,21}}{v_{r,11}}\frac{v_{r,32}}{v_{r,22}}\frac{f_{r,13}}{v_{r,33}}\right)},$$

where

(6) $$\begin{array}{cc} f_{r,11}=\frac{\lambda_{r}}{c}\beta_{\mathrm{DD}}{\bar{F}}_{D} & f_{r,13}=\frac{\lambda_{r}}{c}\beta_{\mathrm{DE}}{\bar{F}}_{D}\frac{\frac{m_{\text{out}}}{\sigma }{\bar{J}}_{E}}{\left({\bar{F}}_{U}+\frac{m_{\text{out}}}{m_{\text{in}}}{\bar{J}}_{E}\right)}\\ \begin{array}{c} f_{r,21}=\frac{\lambda_{r}}{c}\beta_{\mathrm{ED}}{\bar{J}}_{E}\\ v_{r,11}=\mu_{r}+h+\varepsilon_{r}\gamma_{T}\frac{{\bar{L}}_{\mathrm{sD}}}{{\bar{F}}_{D}}\\ v_{r,32}=-m_{\text{out}} \end{array} & \begin{array}{c} f_{r,33}=\frac{\lambda_{r}}{c}\beta_{\mathrm{UU}}\left({\bar{F}}_{U}+\frac{m_{\text{out}}}{m_{\text{in}}}{\bar{J}}_{E}\right)\\ v_{r,22}=m_{\text{out}}+\mu_{r}+\mu_{j}+\alpha +\alpha \frac{{\bar{L}}_{\mathrm{sE}}}{{\bar{J}}_{E}}\\ v_{r,33}=m_{\text{in}}+\mu_{r}+\delta +\delta {\bar{L}}_{\mathrm{sU}}/\left({\bar{F}}_{U}+\frac{m_{\text{out}}}{m_{\text{in}}}{\bar{J}}_{E}\right) \end{array} \end{array}$$

Here, a bar over a variable indicates its nonzero equilibrium level, assumed to exist, in the absence of resistant lice. If *R*_0,r_ > 1, resistant lice will be able to invade, and resistance will spread.

Note that there is a critical host threshold for susceptible lice to persist in the unexposed environment (Frazer et al., 2012). Setting the net reproductive number for susceptible lice, *R*_0,_ *_sW_* (Equation A20) equal to one in the wild‐only model, the critical unexposed wild‐host abundance threshold for louse persistence is:

(7) $$F_{U}^{\text{crit}}=\frac{m_{\text{in}}+\mu_{s}+\delta }{\frac{\lambda_{s}}{c}\beta_{\mathrm{UU}}}-\frac{m_{\text{out}}}{m_{\text{in}}}\left(\frac{a_{0}}{m_{\text{out}}+\mu_{j}}-b\right)$$

### 2.2 Numerical results

We explored the qualitative behaviour of our model by numerically solving System (2) using the *desolve* package in R (R Development Core Team, 2017). Where possible, we fixed parameters at realistic values (given in Table 2), many of which were drawn from sources in the literature that employed comparable modelling frameworks to answer different questions (Frazer et al., 2012; Peacock et al., 2014). In the case of parameters of interest and parameters for which we lacked reasonable estimates, we considered a range of values. In particular, we considered the influence of treatment intensity, *γ_T_*; the unexposed‐host population size, *F_U_*; treatment susceptibility in resistant lice, *ε_r_*; and the cost of resistance, in terms of the mortality rate relative to that for susceptible parasites, *μ_r_*/*μ_s_*. We also considered maximum values and half‐saturation points of the exposed‐juvenile growth rate, *a*_0_ and *b*, respectively as well as louse transfer rates between exposed and domestic hosts, *β*_ED_ and *β*_DE_, and within the unexposed population, *β*_UU_ (full results in Appendix A.6).

**Table 2:** Parameter values

Symbol	Value^a^	Explanation
*F_D_*	6 × 10^6^	Approximate number of farm hosts in the Broughton Archipelago, BC (Frazer et al., 2012)
*F_U_*	( $F_{D}\cdot 20$)	Varied to explore influence of wild‐refuge host population
$\beta_{0,DD}$	1.77×10^−7^	Annualized version of on‐farm daily transfer rate, estimated based on critical production threshold (Frazer et al., 2012)
ψ	0.22	Cumulative survival rate for attached parasite stages preceding adult stage (Frazer et al., 2012; Krkošek, Lewis, & Volpe, 2005)
$\beta_{\mathrm{DD}}$	$\beta_{0,DD}\cdot \psi$	By definition (main text)
$\beta_{\mathrm{DE}}\&\beta_{\mathrm{ED}}$	( $\beta_{\mathrm{DD}}/2$)	Exposed‐host population less dense than domestic population; varied to explore influence of louse transfer between domestic and exposed wild host populations
$\beta_{\mathrm{UU}}$	$\beta_{\mathrm{DD}}/10$	Order‐of‐magnitude approximation; varied to explore influence of unexposed‐host population size
*a*_0_	(5×10^7^)	Varied to explore influence of exposed‐host population features
*b*	(1×10^5^)	varied to explore influence of exposed‐host population features
*m*_out_	4	Juvenile hosts take an average of about three months (=0.25 years=1∕*m_out_*) to migrate to sea (Peacock et al., 2014)
*m*_in_	0.8	adult hosts spend an average of about fifteen months (=1.25 years=1∕*m_in_*) at sea
σ	4	Assume that returning spawners spend an average of about three months ( =0.25years=1/σ) in the coastal environment
*µ_s_*	6.08	Sea lice live an average of about 60 days (=1.67×10^−2^ years $=1/\mu_{s}$; Frazer et al., 2012)
*µ_r_*	( $\mu_{s}\cdot 1.05$)	Varied to explore the cost of resistance suffered by resistant lice
*µ_J_*	$m_{\text{out}}\cdot 19$	Approximately 95% (nineteen in twenty) juvenile salmon die during migration (Heard, 1991; Willette et al., 2001)
*γ_T_*	(25)	Varied to explore influence of treatment strength
*ε_r_*	(0.05)	Varied to explore influence of treatment susceptibility
*h*	0.67	Hosts remain in farms for an average of 1.5 years (=1/*h*)
α	7.3	0.02 hosts killed per parasite per day (Peacock et al., 2014), converted to annual rate
δ	3	Density dependence giving louse abundances in approximate agreement with observed values (Costello, 2006)
$\lambda_{s}$	2.32×10^3^	Approximately 6.35 larvae produced per louse per day (Frazer et al., 2012), converted to annual rate
*c*	73	Larvae survive an average of five days (= 1.34×10^−2^ years =1/c; Frazer et al., 2012)

^a^ Parentheses indicate reference values used to explore the influence of other terms.

In each numerical simulation, unless otherwise noted, we allowed the resistance‐free system to run to approximate equilibrium (5,000 annual time steps), then added a single resistant louse to the domestic environment and allowed the system to again run to approximate equilibrium (usually another 5,000 annual time steps). We refer to the addition of this single resistant louse as “inoculation.”

**Figure 2:**
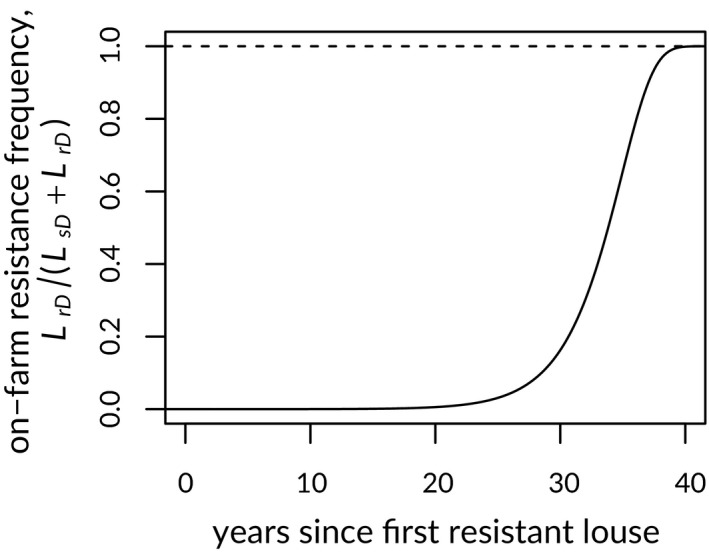
Change in the proportion of lice resistant to treatment (curve) after initial resistance emergence in a differential‐equation model tracking treatment‐resistant and treatment‐susceptible sea louse parasites on farmed (domestic) salmon subject to antilouse treatment. See text for details. Dashed line indicates complete resistance in the domestic sea louse population

To begin, we present the dynamics of resistance in the farm‐only model, using default model parameters, in the absence of wild salmon (for details, see Appendix A.2). In this model, resistance frequency increased approximately logistically after inoculation (Figure 2), reaching nearly 100% after 40 years.

**Figure 3:**
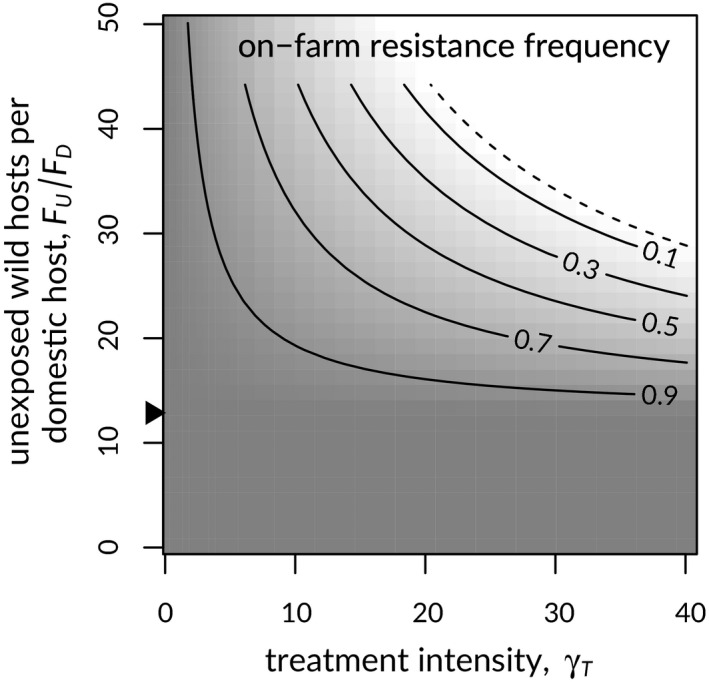
Equilibrium proportion of sea lice resistant to treatment on salmon farms in relation to the rate of treatment increase per louse per domestic host (*γ_T_*) and the size of the unexposed wild host population, relative to the domestic host population (*F_U_*/*F_D_*). The dashed line shows the threshold value of the net reproductive number for resistant lice, *R*_0,_ *_r_ = *1, below which resistance cannot spread. % indicates the threshold unexposed population size required to sustain lice in the system, in the absence of farms (Equation A20)

The equilibrium proportion of lice that were resistant decreased with increasing treatment intensity and unexposed‐host population size (*γ_T_* and *F_U_*, respectively; Figure 3). When both the treatment intensity and the unexposed‐host population size were particularly high, *R*_0,_ *_r_* was less than one, and resistance was unable to invade (Figure 3), in line with the HDR hypothesis. Here, the ability to invade would mean that a small resistant louse population would grow, but without any guarantee that it would grow to comprise all lice in the system.

**Figure 4:**
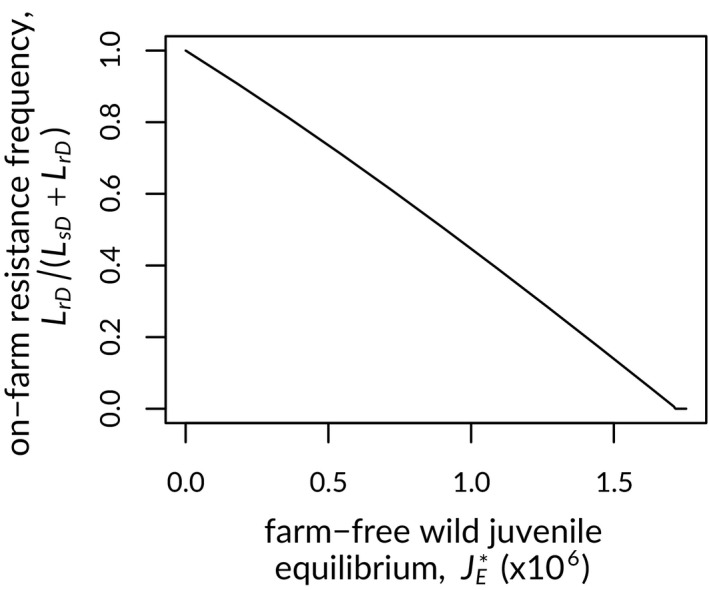
Equilibrium frequency of treatment resistance (curve) in relation to the farm‐free equilibrium abundance of exposed wild juvenile hosts ( $J_{E}^{*}$). Assuming a fixed per capita rate of increase at low population size, $J_{E}^{*}$ is the stable juvenile population size that would result, in a farm‐free model, from varying the maximum population growth rate. See text for details

To explore the critical added feature of our model—the role of wild hosts as vectors connecting louse populations in the treated and untreated environments—we recorded how resistance frequency changed in response to increasing wild‐juvenile population size, *J_E_*. To do this, we numerically solved for the equilibrium frequency of resistance across a range of wild‐juvenile population growth parameters, *a*_0_ and *b*. Here, we assumed the per capita rate of increase at low population size (*a*_0_/*b*) to be a fixed characteristic of wild salmon, and we varied *a*_0_ in simulations, altering *b* to retain the same value of *a*_0_/*b*. We also calculated the equilibrium exposed‐juvenile population sizes ( $J_{E}^{*}$; Equation A15) that would result, given those parameters, in the absence of salmon farms in the system. Population growth parameters that would have produced more “exposed” wild juveniles at equilibrium, prior to the introduction of farms, result in reduced treatment‐resistance frequencies, with very large exposed populations precluding resistance (Figure 4).

In our model, treatment directly reduces parasites in the treated environment, which in turn has the potential to affect connectivity between parasite populations in the treated and untreated environments by reducing the parasite burden on the migratory host vector population. This feature differs from the crop‐pest models in which the HDR strategy was initially identified (Comins, 1977), where pests are able to migrate irrespective of treatment. To explore how treatment affects wild‐host‐mediated connectivity and farm lice in the system, we solved for equilibrium exposed wild‐host population size and on‐farm louse population sizes across a range of treatment intensities, with other parameters held at their default values. We repeated this for versions of the model with and without resistant lice allowed to invade the system, and for a version of the model without any wild hosts present.

**Figure 5:**
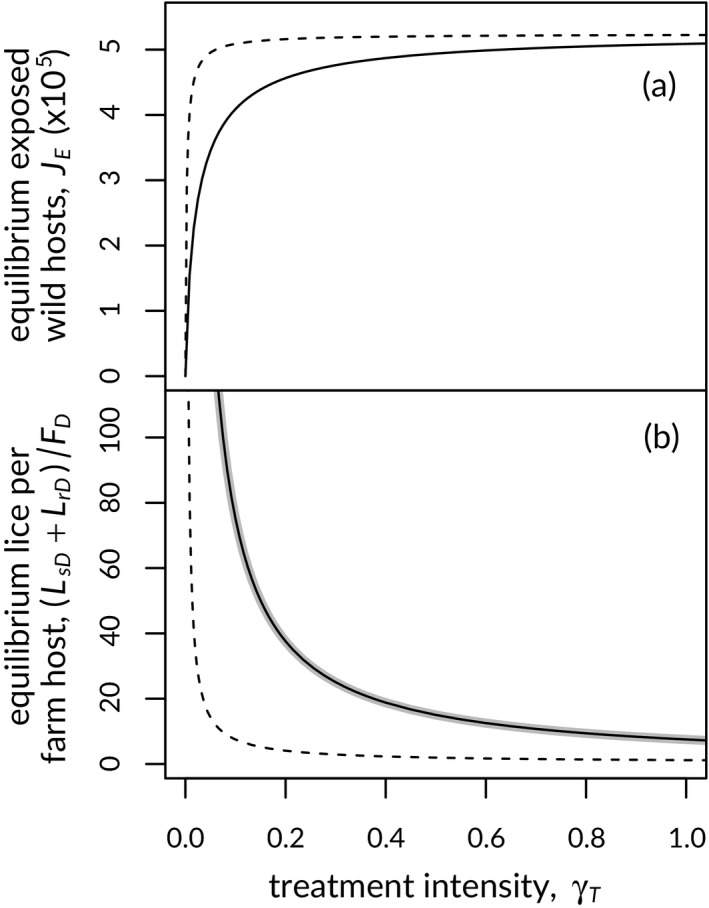
Increased treatment intensity results in larger exposed wild‐host population sizes (a) and fewer total parasites per domestic host (b) at equilibrium. Solid curves show results when resistance is included in the model, and dashed curves show results when the possibility of resistance is omitted from the model. Black curves show results from a model including both farmed and wild hosts, and the thick grey curve shows results from a farm‐only model, without wild hosts. See text for details

Equilibrium levels of wild hosts in the exposed environment increased quickly, and domestic lice numbers declined quickly, as per‐louse treatment intensity, *γ_T_*, increased from zero (Figure 5). By reducing the number of lice in the domestic environment that can spread to exposed wild juveniles, higher treatment intensity serves to protect hosts migrating through the exposed environment, leading to larger exposed‐host populations. The inclusion of resistant lice in the system reduces the protective effect of treatment, leading to more lice in the domestic environment, and results in fewer hosts in the exposed environment at equilibrium. Despite the finding that a larger exposed wild‐host population reduces resistance frequency on farms (Figure 4), the total farm louse population superficially appears little affected by the presence of wild hosts (Figure 5b).

**Figure 6:**
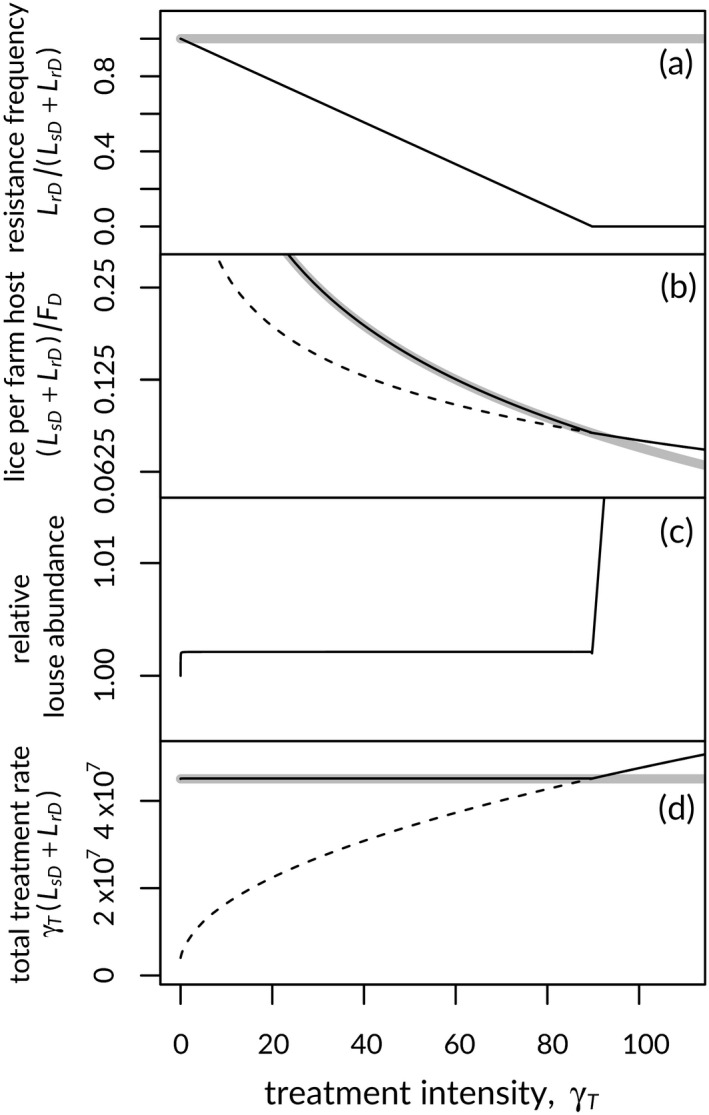
Increased treatment intensity results in reduced on‐farm resistance frequency (a) and fewer lice per farmed host (b; note log scale) at equilibrium. With wild hosts present in the system, the relative number of lice on farms (c), compared to a farm‐only model, is nearly insensitive to treatment intensity up to the level that excludes resistance from the full model at equilibrium. Total treatment rates change little when resistance is present on farms, but rise with increasing treatment intensity when domestic lice are all susceptible to treatment (d). Solid lines in (a), (b) and (d) represent the full model, dashed lines represent a susceptible‐only version of the model, and thick grey lines represent a farm‐only version of the model. See text for details

Further exploring the effects of increased treatment intensity offers insight into mechanisms involved in the observed patterns. After the initial numerical effects on wild hosts and farm resistance frequency (Figure 5), further increases to treatment intensity continue to reduce the resistance frequency in the few lice remaining on farms (Figure 6a). Recall that resistance, in our model, is not complete but controlled by *ε_r_*, so even “resistant” lice are somewhat affected by treatment. At sufficiently high treatment intensity, resistance cannot be sustained at equilibrium (Figure 6a), and after this point, the total number of lice in the domestic environment comes to resemble levels observed when we omit resistance from the model (Figure 6b). Although, up to this point, the total number of lice in the domestic environment resembles that in the farm‐only model, the total louse count is actually slightly higher (Figure 6c), boosted at equilibrium by transmission from wild hosts. The level of similarity between equilibrium domestic louse levels in the full and farm‐only models depends on model parameters (results not shown). Higher levels of lice in the presence of wild hosts result in higher absolute rates of treatment for the same intensity of treatment, elevating the treatment‐induced rate of mortality in resistant lice. The total (as opposed to per‐louse) treatment rate in the domestic environment is higher when resistance is present, than when it is absent, but there is little difference between total treatment rates in the farm‐only model and those in the model that includes wild hosts (Figure 6d). Thus, the addition of wild hosts is able to reduce the level of resistance for a given intensity of treatment, without substantially increasing the number of farm lice or total treatment rate. Also, while the total treatment rate increases with increasing treatment intensity for the (unrealistic) susceptible‐only model, total treatment remains consistent with increasing intensity once resistance is present (Figure 6d).

**Figure 7:**
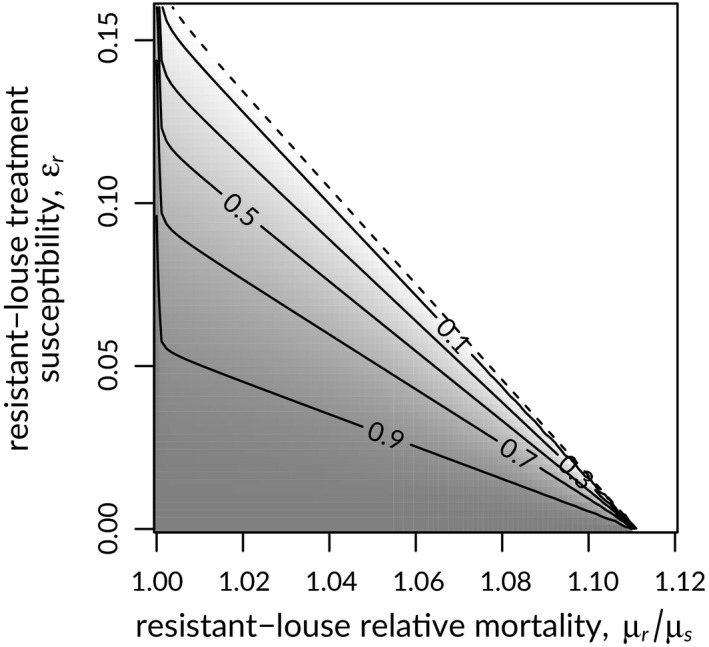
Equilibrium proportion of sea lice resistant to treatment on salmon farms in relation to resistance costs, measured as mortality rate relative to that for susceptible lice (*µ_r_*/*µ_s_*), and resistant louse treatment susceptibility (*ε_r_*). The dashed line in shows the threshold value of the net reproductive number for resistant lice, *R*_0,_ *_r_ = *1, below which resistance cannot spread. See text for details

Increased costs of resistance and treatment susceptibility in resistant lice (*μ_r_*/*μ_s_* and *ε_r_*, respectively) both decreased the equilibrium proportion of lice that were resistant (Figure 7). When costs were high and resistance was incomplete *R*_0,_ *_r_* was less than one, and resistance was unable to invade (Figure 7). If resistance costs were low (1% increase in natural mortality rate), resistant lice could invade if resistance reduced treatment mortality by 85% or more; however, if resistant lice incurred approximately an 11% increase in mortality, resistant lice were unable to invade, even when treatment mortality was extremely low.

## 3 DISCUSSION

Using standard host–macroparasite population models (Anderson & May, 1978), we investigated how ecological factors and management practices could influence the evolution of resistance to chemical treatment in sea louse parasites associated with salmon farms. In our model, the spread of resistant lice within an initially susceptible louse population, inhabiting a domestic host population, was slowed—or stopped—by transmission of lice from a separate wild, untreated host population. This untreated population was unexposed to direct infestation by parasites from the domestic environment. Lice could transmit between the domestic‐ and unexposed‐host populations via a third, migratory host population that reproduced near farms and matured in the unexposed wild environment. Because survival of this connective host population declined with increasing sea louse transfer from salmon farms, treatment on farms proved critical to maintaining the ability of parasites to move from unexposed to domestic environments, promoting treatment susceptibility.

Our results reproduced a “high‐dose/refuge” (HDR) effect, known from the agricultural literature (Comins, 1977; Gould, 1998), whereby high treatment doses combined with immigration of susceptible pests from an untreated refuge population can slow or stop the evolution of resistance in the treated environment (Figure 3). In the classical HDR case, an influx of wild‐type genes from the untreated refuge allows recessive resistant genes to be purged, when subjected to treatment in heterozygous progeny of resident and immigrant pests. Although we instead assumed clonal parasite reproduction and incomplete resistance, our results were similar. Larger unexposed (and untreated) wild‐salmon populations and higher on‐farm treatment rates both reduced the frequency of treatment resistance among lice on farms, with resistance completely precluded at high levels of both treatment and relative unexposed‐host population size (Figure 3; note region with *R*_0,r_ < 1). Unlike the classical HDR effect, the effect in our model arose because parasites from wild hosts slightly increased the parasite burden on domestic hosts, increasing parasite‐dependent treatment rates enough to substantially affect resistance frequencies (Figure 6).

A feature of our model absent from the agricultural literature is the vector population that connects parasite populations in treated and untreated environments. In other models (e.g. Comins, 1977; Murray, 2011), pests migrate on their own between treated and untreated environments. In our model, however, treatment maintained the vector population, which transferred wild‐origin lice to farms, elevating net treatment rates enough to reduce the relative frequency of resistant lice on farms, in a scenario that would otherwise have resulted in resistance (Figure 6). Other studies have shown the importance of a wild refuge in the sea louse/salmon system (Ashander, 2010; Kreitzman et al., 2017; McEwan et al., 2015; Murray, 2011), and at least one study has inferred the importance of exposed wild‐salmon hosts, based on the importance of retaining connectivity between unexposed and farm environments (Kreitzman et al., 2017).

The critical role in our model of migration between distinct host populations underscores the importance of migration and population structure for evolution within host/parasite systems. Migration features in parasite dynamics for animal hosts from ungulates to arthropods (Altizer, Bartel, & Han, 2011; Bartel et al., 2011; Folstad et al., 1991; Mijele et al., 2016), and connectivity among structured host–parasite populations can drive evolution of host defences (Møller & Szép, 2011), parasite virulence (Herre, 1993) and even host speciation (Møller & Szép, 2011). Parasite population structure and connectivity has implications for the effectiveness of antiparasite treatment in other systems. For example, population structure and associated effective population size could have differing effects on the evolution of treatment resistance in helminth parasites of sheep and cattle (Vilas, Vázquez‐Prieto, & Paniagua, 2012), and malarial gene flow, related patterns of antigen diversity and associated selection are likely to affect malaria vaccine design (Barry, Schultz, Buckee, & Reeder, 2009). Building on the known role of pest migration in opposing the evolution of pesticide resistance (Comins, 1977; Huang, Andow, & Buschman, 2011), we have illustrated the role that host migration may play in opposing treatment resistance in parasites.

### 3.1 Assumptions & caveats

Balancing simplicity with tractability and the potential for insight into ecological features of the system, we did not explicitly incorporate population‐genetic considerations (e.g. Ashander, 2010), instead opting for a model that could describe the ecological scenario in a relatively simple way. The model does, however, provide relevant insight, relative to a population‐genetic model with one gene and two alleles (one susceptible and one resistant). Such a population‐genetic model has an *R*_0_, associated with the invasion of a rare heterozygous resistant mutant into a susceptible‐only equilibrium, that is equivalent to our model's *R*_0,r_ under reasonable assumptions (Appendix A.5). Thus, our model provides insight into the potential for a resistance gene, as opposed to a resistant morph, to invade. Of course, the dynamics of the two models would differ if invasion were to occur. For example, we might expect the spread of resistance to accelerate in the diploid case, if the trait were recessive, as resistant homozygotes increased in frequency over time (Hartl & Clark, 2007). In any case, our clonal model, in approximating the invasion behaviour of a single‐gene model of resistance, is most applicable in a high‐treatment‐dose scenario, with selection acting outside the range of standing variation in treatment susceptibility (ffrench‐Constant et al., 2004). In reality, there is some indication that EMB tolerance in Pacific sea lice is polygenic, perhaps indicating incomplete treatment in farmed salmon that are less actively feeding (Sutherland, Elston, & Lambin, 2014). The evolutionary implications of this would likely be better modelled in a quantitative‐genetics framework.

Our three‐environment model (Figure 1) omits geographic complexity associated with wild salmon populations and nearby salmon farms. Salmon farms on the BC coast are geographically clustered, and unlikely to form one homogeneous population. In many cases, sea lice will be much more likely to transfer between localized clusters of salmon farms and nearby wild‐salmon populations than between distant clusters of salmon farms. As a result, treatment resistance may evolve locally and vary geographically. As the abundances of individual wild salmon populations, and their effectiveness as vectors, vary over time (e.g. Decker, Hawkshaw, Patten, Sawada, & Jantz, 2014), selection for or against resistance on clusters of salmon farms is also likely to change. Observed patterns of localized and ephemeral resistance in BC (Messmer et al., 2018; Thomas, 2018) qualitatively match these theoretical predictions. In particular, resistance has tended to emerge in relatively isolated salmon‐farming regions, peripheral to the main clusters of salmon farms on the coast (Messmer et al., 2018). Sea lice in the historical core areas of salmon farming in BC (the Broughton Archipelago and Discovery Islands), through which both local‐ and more distant‐spawning wild salmon populations migrate, have yet to exhibit signs of resistance (Bateman et al., 2016; Saksida et al., 2013). We note, however, that the default parameters we used in simulating our farm‐only model resulted in a substantial resistance increase approximately thirty years after the first resistant louse appeared (Figure 2). This is roughly the same amount of time since the advent of large‐scale salmon farming in Pacific Canada (Ford & Myers, 2008), and we cannot say whether existing conditions may yet lead to widespread resistance in BC.

In addition to geographic complexity, the sea lice/salmon system contains more biological complexity than our model allows. Multiple species of salmonid, with variable life histories (Groot & Margolis, 1991), harbour sea lice in BC. At least one other species of fish, threespine stickleback (*Gasterosteus aculeatus*), can also serve as hosts for part of the sea louse life cycle (Jones, Kim, & Dawe, 2006). Further, we did not consider overdispersion (relative to a Poisson distribution) of sea lice on their hosts. Such overdispersion can arise for a number of reasons and can result in larger equilibrium populations of affected hosts, since more clustered parasites tend to be lost in each parasite‐induced host mortality (Anderson & May, 1978). We might thus expect overdispersion to maintain higher wild salmon populations near salmon farms, serving to better connect populations of sea lice on farmed and wild salmon and further promoting treatment susceptibility in lice. Such considerations could inform future models of EMB resistance in sea lice.

The relative costs and benefits of resistance in our model affected the ability of resistant lice to invade the system as well as the relative equilibrium abundance of resistant lice when they could invade (Figure S2). In our model, some cost of resistance or some susceptibility to treatment in resistant lice was required to avoid the emergence of resistance. In empirical studies, no fitness costs have been observed in sea lice resistant to EMB (Aaen et al., 2015; Espedal et al., 2013). Studies have not, however, compared long‐term performance of resistant and susceptible strains. In our model, a mortality cost of about 11% was necessary in completely resistant lice (Figure 7), but the costs required to preclude resistance decreased almost linearly with increasing treatment susceptibility in resistant lice (Figure 7). Empirical studies have indeed found EMB resistance in sea lice to be incomplete (e.g. Espedal et al., 2013). Even in the absence of high fitness costs, our results suggest that resistance is far from guaranteed in this scenario.

While we considered how the survival benefits and mortality costs of resistance may influence its equilibrium prevalence in a population, we did not explicitly link these parasite characteristics via a trade‐off function. Nor did we consider alternative costs (e.g. to larval development). Such considerations can have important consequences for evolutionary outcomes. For example, in models of parasite virulence evolution, the transmission rate must be an increasing function of virulence, with a negative second derivative, for an optimal level of virulence to be possible (Alizon, Hurford, Mideo, & Baalen, 2009). Different mechanisms of resistance are likely to face different constraints, and the constraints themselves may evolve over time (Andersson, 2006). Understanding such features of resistance trade‐offs presents important avenues for future research.

### 3.2 Relevance for salmon farming

Around the globe, multiple chemicals have been used to control sea lice on farmed salmon (Aaen et al., 2015; Denholm et al., 2002). Although lice have shown reduced sensitivity to many of these drugs in the north Atlantic Ocean (Aaen et al., 2015; Murray, 2011), there has not been widespread resistance to emamectin benzoate (EMB) in Pacific Canada, despite historical reliance on that single drug (Saksida, Morrison, & Revie, 2010, Aaen et al., 2015, Bateman et al., 2016, but see Messmer et al., 2018). While *L. salmonis* in the Atlantic and the Pacific may be different subspecies (Skern‐Mauritzen, Torrissen, & Glover, 2014), the ecology of the two farming regions also differs substantially. In the Atlantic, wild salmon populations are dwarfed by domesticated salmon, but in the north Pacific the presence of large populations of wild hosts is a likely factor in the slow (or patchy) evolution of EMB resistance (Ashander, 2010; Kreitzman et al., 2017; McEwan et al., 2015; Messmer et al., 2018). Our results corroborate the findings that wild‐host abundance may play a role in opposing resistance.

The level of connectivity between louse populations in domestic and unexposed environments was important in determining the emergence of treatment resistance in our model, as reflected in the effect of exposed‐host population size on resistance evolution (Figure 4). Large numbers of exposed hosts, bringing susceptible lice to farms from the unexposed environment, contributed just enough lice to farms to increase louse‐dependent treatment rates, reducing the equilibrium level of resistance or the ability of resistance to evolve in the system (Figure 6). A similar effect occurred when transfer rates between domestic and exposed hosts increased. Higher rates of parasite transmission resulted in slower increase of resistance in lice, lower resistance frequencies at equilibrium and, in extreme cases, the inability of resistance to invade (Figure S4). The connectivity effect of migratory hosts is relevant for management of sea lice on salmon farms because treatment both selects for resistance and protects migratory wild salmon, maintaining the flow of susceptible lice to farms that is needed to slow or stop resistance from evolving. Our model assumes that treatment occurs at some frequency, to reflect current regulatory policies that require treatment or harvesting when louse abundance exceeds a threshold. At extremely low treatment intensities, we would expect the high associated louse loads (Figure 5b) to result in parasite‐induced mortality (or early harvest to avoid such an outcome), reducing domestic‐host abundance and regulating domestic parasite abundance in the process.

Given that treatment does occur, our model supports the perhaps counter‐intuitive past finding that higher levels of treatment should lead to lower levels of resistance (Comins, 1977). In our case, elevating treatment intensity (as opposed to a fixed treatment level) was effective, but only when migrating salmon near to farms can connect those farms to a large untreated host refuge. The prediction that wild‐host populations slightly increase louse burdens on domestic hosts (Figure 6c) is what allows a given treatment intensity to reduce the frequency of resistance, relative to a system without wild hosts (Figures 4 and 6a). This can occur without substantially increasing the total treatment rate (Figure 6d).

Our findings have direct implications for management of sea lice on salmon farms, as two relevant features of the system are under management control: domestic‐salmon stocking levels and treatment responses to infestation levels. Reducing the number of fish on a farm and responding aggressively to louse infestations both appear to reduce the chances of resistance. The importance of intensive treatment to maintain EMB susceptibility in sea lice bears careful consideration in the light of the possibility that variable treatment uptake may have contributed to polygenic resistance in lice (Sutherland et al., 2015). Such indications suggest the importance of carefully monitoring feeding performance in farmed fish.

One critical feature of our model is outside management control: the abundance of unexposed wild hosts. Below a threshold level of unexposed hosts, farm lice invariably evolved resistance (Figure 3). To avoid such a lower unexposed‐host threshold, the unexposed‐host population had to be able to sustain a population of sea lice in the absence of farms. In most salmon‐farming regions, this feature of the system is not in question. In the eastern north Pacific, for example, salmon return from the open ocean with near 100% sea louse prevalence (Gottesfeld et al., 2009), and in the north Atlantic, wild salmon appear to accumulate lice at sea (Jacobsen, 1997).

## 4 CONCLUSIONS

Rather than portraying wild salmon spawning near salmon farms as a costly source of lice on those farms (Gudjonsson 2009), our model highlights the beneficial role that wild salmon may play in opposing the evolution of treatment resistance. This role has been previously identified as an “evosystem service,” a benefit afforded to humans by evolutionary processes in an ecological context (Kreitzman et al., 2017). In this light, by tailoring antiparasite treatments to protect out‐migrating juvenile salmon, the salmon‐farming industry might also be acting in its own best interest. A decade of effective sea louse management on salmon farms shows protection of wild salmon to be possible (Peacock et al., 2013), but recent work suggests that care must be taken to respond to climatic and other ecological changes (Bateman et al., 2016). Our work further suggests that rebuilding salmon stocks could slow or reverse the trend towards treatment resistance observed in some salmon‐farming regions (e.g. Messmer et al., 2018).

Conservation of wild salmon populations is of inherent value to people, economies and ecosystems (Willson, Gende, & Marston, 1998), and our work adds to the mounting body of evidence that suggests it is also likely to be in the interest of the salmon aquaculture industry. As global aquaculture production continues to climb (FAO, 2016), reducing disease‐mediated conflict between domesticated and wild fish populations will be a critical part of maintaining healthy oceans (Krkošek, 2017).

Our findings may also have implications for other host–parasite systems in which parasites are shared between wild and domestic hosts. Our results indicate that wild migratory hosts need not merely be a source of infection (Daszak et al., 2000; Rhyan & Spraker, 2010), but may also aid in parasite management in domestic hosts. With wild migratory species particularly at risk from human impacts (Bairlein, 2016; Harris, Caillaud, Chapman, & Vigilant, 2009), this scenario presents the prospect of another reason to conserve such species when they share parasites with domestic species (e.g. Mijele et al., 2016; Morgan, Medley, Torgerson, Shaikenov, & Milner‐Gulland, 2007).

## CONFLICT OF INTEREST

None declared.

## Supporting information


**Appendix S1.**

## Data Availability

Data sharing is not applicable to this article as no new data were created or analysed in this study. Code to simulate the basic differential‐equation model can be found at https://github.com/andrew‐bateman/SLICE‐resistance
