# Supplementary material for: Migratory hosts can maintain the high‐dose/refuge effect in a structured host‐parasite system: The case of sea lice and salmon
Source: Evol Appl. 2020 Sep 10;13(10):2521–35. doi: 10.1111/eva.12984 (PMC7691465; doi:10.1111/eva.12984)
Supplement: Supplementary file 1 — Appendix S1. [file EVA-13-2521-s001.pdf]

Migratory hosts can maintain the high-dose refuge effect in a structured host-parasite system: the case of sea lice and salmon

Andrew W. Bateman, Stephanie J. Peacock, Martin Krkošek, and Mark A. Lewis

Appendix A: Model details

A.1 Details of nonlinear parasite mortality

With two types of lice (susceptible and resistant), louse mortality that results from parasite-induced host mortality in wild juveniles is more complicated than in the standard Anderson-May (1978) host-parasite model. Let $p(i)$ be the probability that a randomly selected host harbours i susceptible parasites, and let $q(k)$ be the independent probability that the host harbours k resistant parasites, so that $p(i)q(j-i)$ is the probability of a host having j total lice, of which i are susceptible. Elaborating the Anderson-May formulation, this mortality rate for susceptible lice, assuming host mortality is linearly dependent on total lice load on a host, and summing across all total lice loads, j, and susceptible lice loads, i:

$$\begin{aligned} &F_X \sum_{j=0}^{\infty} \sum_{i=0}^{j} \alpha j \cdot i \cdot p(i)q(j-i) \\ &= \alpha F_X \sum_{j=0}^{\infty} \sum_{i=0}^{j} ijp(i)q(j-i) \\ &= \alpha F_X \left[\sum_{j=0}^{\infty} \sum_{i=0}^{j} i(j-i)p(i)q(j-i) + \sum_{j=0}^{\infty} \sum_{i=0}^{j} i^2 p(i)q(j-i) \right], \end{aligned} \tag{A1}$$

and after a change of summation indices, this component rate becomes:

$$\begin{aligned} &\alpha F_X \left[\sum_{i=0}^{\infty} \sum_{k=0}^{\infty} ikp(i)q(k) + \sum_{i=0}^{\infty} \sum_{k=0}^{\infty} i^2 p(i)q(k) \right] \\ &= \alpha F_X \left[E_p(i)E_q(k) + E_p(i^2) \right]. \end{aligned} \tag{A2}$$

If we assume that lice are Poisson-distributed, then $E_p(i^2) = [E_p(i)]^2 + E_p(i)$, and the rate of parasite mortality due to parasite-induced host mortality is:

$$\begin{aligned}
&\alpha F_X E_p(i)\left[E_q(k) + E_p(i) + 1\right] \\
&\quad = \alpha F_X \frac{L_{sX}}{F_X}\left(\frac{L_{rX}}{F_X} + \frac{L_{sX}}{F_X} + \frac{F_X}{F_X}\right) \\
&\quad = \alpha L_{sX}\left(\frac{L_{rX} + L_{sX} + F_X}{F_X}\right)
\end{aligned} \tag{A3}$$

A.2 Model equations

Starting with host-parasite dynamics on farms:

$$\frac{dF_D}{dt} = g - h \qquad (F_D \text{ constant}) \tag{A4a}$$

$$\frac{dL_{sD}}{dt} = \left(\beta_{DD} C_{sD} + \beta_{DE} C_{sS}\right) F_D - (\mu_s + h) L_{sD} - \mu_{sT} L_{sD} \tag{A4b}$$

$$\frac{dL_{rD}}{dt} = \left(\beta_{DD} C_{rD} + \beta_{DE} C_{rS}\right) F_D - (\mu_r + h) L_{rD} - \mu_{rT} L_{rD}, \tag{A4c}$$

where C_{yX} represents copepodid larvae produced by the appropriate parasite populations (addressed below), and C_{yS} represents copepodid larvae produced by adult lice on the exposed-origin adult host population returning to spawn (L_{yS}). Here, host fish are stocked louse-free, but we assume that the stocking and harvest rates are equal, so that $dF_D/dt = 0$. We assume the treatment mortality rates, μ_{sT} and μ_{rT}, are functions of the average louse load per domestic host, $\left(L_{rD} + L_{sD}\right)/F_D$, and the effectiveness of treatment on the given class of parasite (susceptible or resistant). Assuming a linear relationship between louse abundance and treatment level, we can write:

$$\mu_{xT} = \varepsilon_y \gamma_T \left(\tfrac{L_{rD} + L_{sD}}{F_D}\right), \tag{A5a}$$

where ε_y is the treatment effectiveness for louse class y, and γ_T is the increase in treatment mortality rate on farms for each additional louse per host. We define ε_s to be one, so that ε_r controls the sensitivity of resistant lice to treatment.

Taking domestic resistant copepodid lice as an example case, copepodid dynamics are given

by:

$$\frac{dC_{rD}}{dt} = \lambda_r L_{rD} - cC_{rD} - \beta_{0,ED} C_{rD} J_E - \beta_{0,DD} C_{rD} F_D. \tag{A6}$$

Ignoring lice of wild origin, our model of on-farm host-parasite dynamics is thus equivalent to that used by Frazer et al. (2012), with the exception that we model infestation-responsive (rather than constant) treatment levels. Assuming copepodid dynamics are fast, relative to host-parasite dynamics in general (May and Anderson 1979), $dC_{rD}/dt = 0$ and copepodid abundance is given by:

$$C_{rD} = \frac{\lambda_r L_{rD}}{c + \beta_{0,ED} J_E + \beta_{0,DD} F_D}. \tag{A7}$$

Other copepodid abundances take similar forms. We assume that copepodid attachment is highly unlikely, so that $c >> \beta_{0,X_2X_1}$. In this case, $C_{yX} = \lambda_y L_{yX}/c$.

For the exposed host population, we assume that juveniles are susceptible to parasite-induced mortality, while adults are not. Juvenile exposed hosts pick up lice from the domestic environment, suffer associated mortality, and then mature as they migrate to the environment shared with unexposed hosts during adulthood. When adult exposed hosts return as spawners, S_E, to spawn near the domestic environment, they bring parasites from the shared wild environment and give rise to new juvenile exposed hosts (assuming density-dependent population growth). We assume parasites do not have time to reproduce on exposed juvenile hosts, before they are carried to the unexposed wild environment, so only domestic-origin copepodid larvae contribute to the exposed parasite population.

$$\frac{dJ_E}{dt} = \frac{a_0 S_E}{b_0 + S_E} - (m_{out} + \mu_J) J_E - \alpha (L_{sE} + L_{rE}) \tag{A8a}$$

$$\frac{dA_U}{dt} = m_{out} J_E - m_{in} A_U \tag{A8b}$$

$$\frac{dS_E}{dt} = m_{in} A_U - \sigma S_E \tag{A8c}$$

$$\frac{dL_{sE}}{dt} = \beta_{ED} C_{sD} J_E - (m_{out} + \mu_s + \mu_J + \alpha) L_{sE} - \alpha L_{sE} \left(\frac{L_{rE} + L_{sE}}{J_E} \right) \tag{A8d}$$

$$\frac{dL_{rE}}{dt} = \beta_{ED} C_{rD} J_E - (m_{out} + \mu_r + \mu_J + \alpha) L_{rE} - \alpha L_{rE} \left(\frac{L_{rE} + L_{sE}}{J_E} \right) \tag{A8e}$$

Copepodid larvae produced by adult lice carried from the unexposed environment on exposed-

origin spawning adult salmon take a slightly different form. We assume that there is not time for lice to reproduce on spawners from the exposed wild population, so that the associated louse populations, L_{yS}, obey dynamics:

$$\frac{dL_{yS}}{dt} = m_{in} L_{yU} \left(\frac{A_U}{F_U + A_U} \right) - \sigma L_{yS}. \tag{A9}$$

Again, assuming fast spawning dynamics, we can solve for the associated equilibrium: $L_{yS} = m_{in} L_{yU} A_U / (\sigma [F_U + A_U])$. As with the other copepodid equations, we assume that copepodid dynamics are fast and mortality overwhelms attachment. Thus,

$$C_{yS} = \frac{\lambda_y}{c} L_{yU} \frac{m_{in}}{\sigma} \frac{A_U}{F_U + A_U}. \tag{A10}$$

As with exposed-origin hosts, we assume that the adult hosts in the unexposed environment suffer no parasite-induced mortality. We do not consider the dynamics of the unexposed host population, as we assume these adult hosts to be unaffected by louse abundance, and dynamical changes to unexposed host population size are therefore outside the scope of this model. The unexposed host population size serves as a parameter in the final model. Parasites in the unexposed environment are hosted by unexposed-origin hosts and adult exposed-origin hosts.

$$\frac{dF_U}{dt} = 0 \qquad (F_U \text{ constant}) \tag{A11a}$$

$$\frac{dL_{sU}}{dt} = \beta_{UU} C_{sU} (F_U + A_U) + m_{out} L_{sE} - (m_{in} + \mu_s + \delta) L_{sU} - \delta L_{sU} \left(\frac{L_{rU} + L_{sU}}{F_U + A_U} \right) \tag{A11b}$$

$$\frac{dL_{rU}}{dt} = \beta_{UU} C_{rU} (F_U + A_U) + m_{out} L_{rE} - (m_{in} + \mu_r + \delta) L_{rU} - \delta L_{rU} \left(\frac{L_{rU} + L_{sU}}{F_U + A_U} \right) \tag{A11c}$$

The unexposed hosts may be optional in the model, depending on assumptions. BC might best be modelled with large unexposed populations, while other jurisdictions might best be modelled with no unexposed populations. In the latter case, all wild hosts would be considered to spawn in proximity to the domestic population, with juveniles exposed to domestic-origin lice.

We make the assumption that resistance evolution takes place over longer timescales than dynamics within the exposed host population. That is, we assume the number of exposed adults and spawners equilibriate quickly, and we replace A_U with its equilibrium value, $m_{out} J_E / m_{in}$, and S_E with its equilibrium value, $m_{in} A_U / \sigma = m_{out} J_E / \sigma$.

A.3 Assembled model

From above, we have sets of equations that describe host-parasite and migration dynamics across three environments. We are interested in the dynamics of resistant as compared to non-resistant parasites, in response to treatment in the domestic environment. Exposed-origin hosts function as parasite vectors between the domestic and unexposed wild environments and host-parasite dynamics on exposed juvenile hosts determine vector strength. In our model analyses (below), we seek insight about the system overall but begin by considering simpler sub-components.

farm-only model

First, we consider dynamics in the domestic environment, without any connection to wild fish. This situation could arise in a region without wild salmonids or if local wild salmonids had been extirpated. The model is given by:

$$\frac{dF_D}{dt} = 0 \qquad (F_D \text{ constant}) \tag{A12a}$$

$$\frac{dL_{sD}}{dt} = \frac{\lambda_s}{c}\beta_{DD}L_{sD}F_D - (\mu_s + h)L_{sD} - \gamma_T\left(\tfrac{L_{rD}+L_{sD}}{F_D}\right)L_{sD} \tag{A12b}$$

$$\frac{dL_{rD}}{dt} = \frac{\lambda_r}{c}\beta_{DD}L_{rD}F_D - (\mu_r + h)L_{rD} - \varepsilon_r\gamma_T\left(\tfrac{L_{rD}+L_{sD}}{F_D}\right)L_{rD}. \tag{A12c}$$

wild-only model

Next, we consider the wild system in isolation, without salmon farms. In this case, we only consider louse dynamics in the unexposed, oceanic environment. Although lice do infect juveniles in the absence of salmon farms, infection rates are low (Krkošek et al. 2005), and lice on wild juveniles are strongly associated with the presence of farms (Morton and Williams 2003; Krkošek et al. 2005). Our models do not consider this background infection, focusing instead on the main features of the system, so that lice on exposed hosts would decline to zero without domestic-origin inputs (see equation A8). Replacing $\sigma b_0/m_{out}$ with b, for simplicity, the wild-only model is given by:

$$\frac{dJ_E}{dt} = \frac{a_0 J_E}{b + J_E} - (m_{out} + \mu_J)\, J_E \tag{A13a}$$

$$\frac{dF_U}{dt} = 0 \qquad (F_U \text{ constant}) \tag{A13b}$$

$$\frac{dL_{sU}}{dt} = \frac{\lambda_s}{c}\beta_{UU} L_{sU}\left(F_U + \tfrac{m_{out}}{m_{in}} J_E\right) - (m_{in} + \mu_s + \delta) L_{sU} - \delta \frac{L_{rU} + L_{sU}}{\left(F_U + \frac{m_{out}}{m_{in}} J_E\right)} L_{sU} \tag{A13c}$$

$$\frac{dL_{rU}}{dt} = \frac{\lambda_r}{c}\beta_{UU} L_{rU}\left(F_U + \tfrac{m_{out}}{m_{in}} J_E\right) - (m_{in} + \mu_r + \delta) L_{rU} - \delta \frac{L_{rU} + L_{sU}}{\left(F_U + \frac{m_{out}}{m_{in}} J_E\right)} L_{rU} \tag{A13d}$$

full model

804 Finally, we consider the full model, in which exposed wild hosts serve as the vector connecting the domestic and unexposed wild host populations:

$$\frac{dF_D}{dt} = 0 \tag{A14a}$$

$$\frac{dL_{sD}}{dt} = \frac{\lambda_s}{c}\left[\beta_{DD} L_{sD} + \beta_{DE} L_{sU} \frac{\frac{m_{out}}{\sigma} J_E}{\left(F_U + \frac{m_{out}}{m_{in}} J_E\right)}\right] F_D - (\mu_s + h) L_{sD} - \gamma_T \left(\frac{L_{rD} + L_{sD}}{F_D}\right) L_{sD} \tag{A14b}$$

$$\frac{dL_{rD}}{dt} = \frac{\lambda_r}{c}\left[\beta_{DD} L_{rD} + \beta_{DE} L_{rU} \frac{\frac{m_{out}}{\sigma} J_E}{\left(F_U + \frac{m_{out}}{m_{in}} J_E\right)}\right] F_D - (\mu_r + h) L_{rD} - \varepsilon_r \gamma_T \left(\frac{L_{rD} + L_{sD}}{F_D}\right) L_{rD} \tag{A14c}$$

$$\frac{dJ_E}{dt} = \frac{a_0 J_E}{b + J_E} - (m_{out} + \mu_J)\, J_E - \alpha\,(L_{sE} + L_{rE}) \tag{A14d}$$

$$\frac{dL_{sE}}{dt} = \frac{\lambda_s}{c}\beta_{ED} L_{sD} J_E - (m_{out} + \mu_s + \mu_J + \alpha)\, L_{sE} - \alpha\left(\frac{L_{rE} + L_{sE}}{J_E}\right) L_{sE} \tag{A14e}$$

$$\frac{dL_{rE}}{dt} = \frac{\lambda_r}{c}\beta_{ED} L_{rD} J_E - (m_{out} + \mu_r + \mu_J + \alpha)\, L_{rE} - \alpha\left(\frac{L_{rE} + L_{sE}}{J_E}\right) L_{rE} \tag{A14f}$$

$$\frac{dF_U}{dt} = 0 \tag{A14g}$$

$$\frac{dL_{sU}}{dt} = \frac{\lambda_s}{c}\beta_{UU} L_{sU}\left(F_U + \tfrac{m_{out}}{m_{in}} J_E\right) + m_{out} L_{sE} - (m_{in} + \mu_s + \delta) L_{sU} - \delta \frac{L_{rU} + L_{sU}}{\left(F_U + \frac{m_{out}}{m_{in}} J_E\right)} L_{sU} \tag{A14h}$$

$$\frac{dL_{rU}}{dt} = \frac{\lambda_r}{c}\beta_{UU} L_{rU}\left(F_U + \tfrac{m_{out}}{m_{in}} J_E\right) + m_{out} L_{rE} - (m_{in} + \mu_r + \delta) L_{rU} - \delta \frac{L_{rU} + L_{sU}}{\left(F_U + \frac{m_{out}}{m_{in}} J_E\right)} L_{rU} \tag{A14i}$$

A.4 Model analysis

A.4.1 Equilibria

For the full model of the system, (A14), the louse-free nonzero equilibrium, $\{F_D^*, J_E^*, F_U^*\}$ results from setting dJ_E/dt and all louse abundances equal to zero (dF_D/dt and dF_E/dt are zero by definition):

$$\frac{dJ_E}{dt} = 0 \Rightarrow$$
$$J_E^* = \frac{a_0}{m_{out} + \mu_j} - b. \tag{A15}$$

$J_E = 0$, the trivial equilibrium, also satisfies $dJ_E/dt = 0$. F_D^* and F_U^* are arbitrary constants.

In the absence of lice, the positive equilibrium abundances for systems (A12) and (A13) are simply subsets of $\{F_D^*, J_E^*, F_U^*\}$. The farm-only system has louse-free equilibrium $\{F_D^*\}$, and the wild-only system has equilibrium $\{J_E^*, F_U^*\}$.

farm-only model

When only susceptible lice are present, system (A12) has nontrivial equilibrium $\{F_D^{**}, L_{sD}^{**}\}$. Setting $dL_{sD}/dt = 0$ in equation (A12), $L_{sD}^{**} = (\frac{\lambda_s}{c}\beta_{DD}F_D^{**} - \mu_s - h)F_D^{**}/\gamma_T$. Because $F_D^{**} = F_D^*$ is arbitrary, the susceptible-lice equilibrium can be written in terms of the value of the louse-free host abundance, F_D^*, above.

wild-only model

For the wild-host system, (A13), we can also set $F_U^{**} = F_U^*$ arbitrarily. Solving $dJ_E/dt = 0$ and $dL_{sU}/dt = 0$ in equation (A12), $J_E^{**} = J_E^*$ and $L_{sU}^{**} = F_W^*(\frac{\lambda_s}{c}\beta_{UU}F_W^* - m_{in} + \mu_s + \delta)/\delta$, where $F_W^* = F_U^* + \frac{m_{out}}{m_{in}}J_E^*$ (total wild adult hosts at equilibrium in the absence of farms).

full model

When susceptible lice are present, rearranging equations (A14) provides conditions for the equilibrium, $\{\overline{F}_D, \overline{L}_{sD}, \overline{J}_E, \overline{L}_{sE}, \overline{F}_U, \overline{L}_{sU}\}$. Once again, $\overline{F}_D = F_D^*$ and $\overline{F}_U = F_U^*$ are arbitrary. The associated nullclines are given by:

$$
\begin{aligned}
\frac{dL_{sD}}{dt} &= 0 \Rightarrow \\
0 &= \tfrac{\gamma_T}{\overline{F}_D}\Big(F_U + \tfrac{m_{out}}{m_{in}}J_E\Big)\overline{L}_{sD}^{\,2} + (\mu_s + h - \tfrac{\lambda_s}{c}\beta_{DD}\overline{F}_D)\Big(F_U + \tfrac{m_{out}}{m_{in}}J_E\Big)\overline{L}_{sD} \\
&\quad - \tfrac{\lambda_s}{c}\beta_{DE}\overline{F}_D\tfrac{m_{out}}{\sigma}\overline{J}_E\overline{L}_{sU} && \text{(A16a)} \\
\frac{dJ_E}{dt} &= 0 \Rightarrow \\
0 &= \left(\overline{J}_E - \left[\frac{a_0}{m_{out} + \mu_j} - b\right]\right)\overline{J}_E + \frac{\alpha}{m_{out} + \mu_j}\overline{L}_{sE}\overline{J}_E + \frac{\alpha b}{m_{out} + \mu_j}\overline{L}_{sE} && \text{(A16b)} \\
\frac{dL_{sE}}{dt} &= 0 \Rightarrow \\
0 &= \alpha\overline{L}_{sE}^{\,2} + (m_{out} + \mu_s + \mu_j + \alpha)\overline{L}_{sE}\overline{J}_E - \tfrac{\lambda_s}{c}\beta_{ED}\overline{L}_{sD}\overline{J}_E^{\,2} && \text{(A16c)} \\
\frac{dL_{sU}}{dt} &= 0 \Rightarrow \\
0 &= \delta\overline{L}_{sU}^{\,2} + [m_{in} + \mu_s + \delta - \tfrac{\lambda_s}{c}\beta_{UU}(\overline{F}_U + \tfrac{m_{out}}{m_{in}}\overline{J}_E)](\overline{F}_U + \tfrac{m_{out}}{m_{in}}\overline{J}_E)\overline{L}_{sU} \\
&\quad - m_{out}(\overline{F}_U + \tfrac{m_{out}}{m_{in}}\overline{J}_E)\overline{L}_{sE}. && \text{(A16d)}
\end{aligned}
$$

The nullclines for the full model are even more complicated, and we do not show them here.

A.4.2 Net reproductive ratios

Here, we assume that positive (nontrivial) equilibria exist for the various systems we consider.

In epidemiology, the net reproductive ratio, R_0, typically measures the number of new cases of a disease produced by a single initial infection in a population of susceptible individuals (Heesterbeek 2002; van den Driessche and Watmough 2002). In our context, R_0 measures the number of parasites produced by an average parasite in the system, over the course of its life, when parasites are rare. R_0 is a useful quantity to compute, indicating the potential for invasion ($R_0 > 1$) or clearance

($R_0 < 1$) of the parasite population (van den Driessche and Watmough 2002).

farm-only model

In general terms, R_0 equals a pathogen's reproductive rate times its average lifespan in the system (the inverse of its mortality rate or rate of efflux from the system), when rare. This is straightforward to calculate for the domestic louse populations in isolation (Heesterbeek 2002). For susceptible lice in an initially louse-free domestic environment:

$$\begin{aligned} \frac{dL_{sD}}{dt} &= \frac{\lambda_s}{c}\beta_{DD}L_{sD}F_D - (\mu_s + h)L_{sD} - \gamma_T\frac{L_{sD}}{F_D}L_{sD} \\ \Rightarrow R_{0,sD} &= \left.\frac{\frac{\lambda_s}{c}\beta_{DD}F_D^*}{\mu_s + h + \gamma_T L_{sD}/F_D^*}\right|_{L_{sD}=0} \\ &= \frac{\frac{\lambda_s}{c}\beta_{DD}F_D^*}{\mu_s + h}. \end{aligned} \tag{A17}$$

We assume that $R_{0,sD} > 1$, given that lice proliferate on farms in that absence of treatment.

For resistant lice, we consider invasion in the context of evolution. We want to know if a small number of mutant resistant lice will proliferate when introduced into a stable system of hosts and susceptible lice. For the isolated domestic environment, we consider the equilibrium $\{F_D^{**}, L_{sD}^{**}\}$, described above. As with $R_{0,sD}$, R_0 for the resistant lice is straightforward to calculate:

$$\begin{aligned} R_{0,rD} &= \frac{\frac{\lambda_r}{c}\beta_{DD}F_D^{**}}{\mu_r + h + \epsilon_r\gamma_T L_{sD}^{**}/F_D^{**}} \\ &= \frac{\frac{\lambda_r}{c}\beta_{DD}F_D^*}{\mu_r + h + \epsilon_r(\frac{\lambda_s}{c}\beta_{DD}F_D^* - \mu_s - h)}. \end{aligned} \tag{A18}$$

Assuming on-farm louse production exceeds louse mortality and harvest rates,

$$R_{0,rD} > 1 \iff \epsilon_r < \frac{\frac{\lambda_r}{c}\beta_{DD}F_D^* - \mu_r - h}{\frac{\lambda_s}{c}\beta_{DD}F_D^* - \mu_s - h}. \tag{A19}$$

That is, in the absence of external influences, resistant lice could invade farms whenever treatment susceptibility of resistant lice is lower than their proportional growth rate (relative to susceptible lice) at low louse abundance. We assume this is the case, given that we are concerned with situations in which resistant lice have the potential to proliferate in the farm environment. We

note that as the domestic host population increases, louse mortality and harvest rates have less impact. Also, given our assumption that treatment rates depend on the average number of lice per farmed host, treatment intensity has no bearing on the ability of resistant lice to invade an isolated farm system.

wild-only model

For the wild population in the absence of farms, described by system (A13), the R_0 calculations are similar. Although there are two classes of hosts (exposed and unexposed), we have assumed that there are no lice on "exposed" juvenile hosts in the absence of farms. Considering invasion of the initially louse-free system,

$$R_{0,sW} = \frac{\frac{\lambda_s}{c}\beta_{UU}F_W^*}{m_{in} + \mu_s + \delta}. \tag{A20}$$

As sea lice are a ubiquitous component of wild salmonid populations around the world, we assume below that susceptible lice can invade the parasite-free system, so that $R_{0,sW} > 1$.

For resistant lice invading the wild-only host population at equilibrium with susceptible lice,

$$\begin{aligned} R_{0,rW} &= \frac{\frac{\lambda_r}{c}\beta_{UU}F_W^{**}}{m_{in} + \mu_r + \delta + \delta L_{sU}^{**}/F_W^{**}} \\ &= \frac{\frac{\lambda_r}{c}\beta_{UU}F_W^{*}}{\mu_r - \mu_s + \frac{\lambda_s}{c}\beta_{UU}F_W^{*}}. \end{aligned} \tag{A21}$$

Here, we see that the ability of resistant lice to invade a wild-only adult salmon population depends on the larval production and natural mortality rates of susceptible and resistant lice. Resistant lice may suffer physiological costs of resistance, resulting in $\lambda_r \leqslant \lambda_s$, $\mu_r \geqslant \mu_s$, and $R_{0,rW} \leqslant 1$.

full model

To consider R_0 values for the three-environment system, (A14), we must consider production and mortality of lice in each environment as well as transfer between the environments. Van den Driesche and Watmough (2002) provide a clear methodology for calculation of R_0 values in such situations. To consider initial invasion by susceptible lice, we first define two matrices, $\mathbf{F} = [f_{ij}]$ and

$\mathbf{V} = [v_{ij}]$. $\mathbf{F}$ and $\mathbf{V}$ are both composed of coefficients from the linearisation of system (A14) about the equilibrium without the louse type of interest. Specifically, they come from $[\mathbf{F} - \mathbf{V}]$, the matrix of terms from the linearisation that are associated with the louse type of interest. $\mathbf{F}$ contains the set of coefficients associated with production of new lice, and $\mathbf{V}$ contains the set of terms associated with mortality and transfer of lice. Note that the sign of terms in $\mathbf{V}$ is opposite to that with which they appear in the standard linearisation, so that mortality terms are positive and immigration terms are negative. Once the $\mathbf{F}$ and $\mathbf{V}$ matrices have been constructed, $R_0 = \rho_1[\mathbf{F}\mathbf{V}^{-1}]$ (van den Driessche and Watmough 2002), where $\rho_1[\cdot]$ denotes the dominant eigenvalue of a matrix. For details see van den Driessche and Watmough (2002).

For susceptible lice invading the three-environment louse-free equilibrium, $\{F_D^*, J_E^*, F_U^*\}$,

$$\mathbf{F}_s = \begin{bmatrix} f_{s,11} & 0 & f_{s,13} \\ f_{s,21} & 0 & 0 \\ 0 & 0 & f_{s,33} \end{bmatrix}, \tag{A22}$$

where

$$\begin{array}{ll} f_{s,11} = \frac{\lambda_s}{c}\beta_{DD}F_D^* & f_{s,13} = \frac{\lambda_s}{c}\beta_{DE}F_D^*\frac{m_{out}}{\sigma}\frac{J_E^*}{F_W^*} \\ f_{s,21} = \frac{\lambda_s}{c}\beta_{ED}J_E^* & f_{s,33} = \frac{\lambda_s}{c}\beta_{UU}F_W^* \end{array} \tag{A23}$$

(note the β_{DE} coefficient in the $f_{s,13}$ term, that arises because lice from the unexposed environment are carried by migrating hosts to the exposed environment, from where infestation of domestic hosts occurs), and

$$\mathbf{V}_s = \begin{bmatrix} v_{s,11} & 0 & 0 \\ 0 & v_{s,22} & 0 \\ 0 & v_{s,32} & v_{s,33} \end{bmatrix}, \tag{A24}$$

where

$$\begin{array}{ll} v_{s,11} = \mu_s + h & v_{s,22} = m_{out} + \mu_s + \mu_j + \alpha \\ v_{s,32} = -m_{out} & v_{s,33} = m_{in} + \mu_s + \delta. \end{array} \tag{A25}$$

Applying the R_0 formula (van den Driessche and Watmough 2002):

$$
\begin{aligned}
R_{0,s} &= \frac{1}{2}\left(\frac{f_{s,11}}{v_{s,11}} + \frac{f_{s,33}}{v_{s,33}}\right) + \sqrt{\left[\frac{1}{2}\left(\frac{f_{s,11}}{v_{s,11}} + \frac{f_{s,33}}{v_{s,33}}\right)\right]^2 - \left(\frac{f_{s,11}}{v_{s,11}}\frac{f_{s,33}}{v_{s,33}} + \frac{f_{s,21}}{v_{s,11}}\frac{v_{s,32}}{v_{s,22}}\frac{f_{s,13}}{v_{s,33}}\right)} \\
&= \frac{1}{2}\left(R_{0,sD} + R_{0,sW}\right) + \sqrt{\left[\frac{1}{2}\left(R_{0,sD} - R_{0,sW}\right)\right]^2 - \frac{f_{s,21}}{v_{s,11}}\frac{v_{s,32}}{v_{s,22}}\frac{f_{s,13}}{v_{s,33}}}\,. \qquad \text{(A26)}
\end{aligned}
$$

Note that in addition to R_0 values for the domestic and wild environments in isolation, $R_{0,s}$ contains another term, $f_{s,21}v_{s,32}f_{s,13}/(v_{s,11}v_{s,22}v_{s,33})$. This additional term represents lice transferring among environments; *e.g.* $f_{s,21}/v_{s,11}$ is the number of adult lice on exposed juveniles produced by an average louse in the farm environment over the course of its life. Since all the components of this term are positive, except for $v_{s,32}$, the quantity inside the radical is positive (the other component is squared), and $R_{0,s} > (R_{0,sD} + R_{0,sW})/2$. Since we have assumed $R_{0,sD}$ and $R_{0,sW}$ to be greater than one, $R_{0,s} > 1$. Thus, susceptible lice will invade the louse-free host system at equilibrium.

For resistant lice invading the nontrivial host/susceptible-lice equilibrium, $\{\overline{F}_D, \overline{L}_{sD}, \overline{J}_E, \overline{L}_{sE}, \overline{F}_U, \overline{L}_{sU}\}$, the R_0 calculation is similar:

$$
R_{0,r} = \frac{1}{2}\left(\frac{f_{r,11}}{v_{r,11}} + \frac{f_{r,33}}{v_{r,33}}\right) + \sqrt{\left[\frac{1}{2}\left(\frac{f_{r,11}}{v_{r,11}} + \frac{f_{r,33}}{v_{r,33}}\right)\right]^2 - \left(\frac{f_{r,11}}{v_{r,11}}\frac{f_{r,33}}{v_{r,33}} + \frac{f_{r,21}}{v_{r,11}}\frac{v_{r,32}}{v_{r,22}}\frac{f_{r,13}}{v_{r,33}}\right)}, \qquad \text{(A27)}
$$

where

$$
\begin{aligned}
f_{r,11} &= \tfrac{\lambda_r}{c}\beta_{DD}\overline{F}_D & f_{r,13} &= \tfrac{\lambda_r}{c}\beta_{DE}\overline{F}_D\frac{\frac{m_{out}}{\sigma}\overline{J}_E}{\left(\overline{F}_U + \frac{m_{out}}{m_{in}}\overline{J}_E\right)} \\
f_{r,21} &= \tfrac{\lambda_r}{c}\beta_{ED}\overline{J}_E & f_{r,33} &= \tfrac{\lambda_r}{c}\beta_{UU}(\overline{F}_U + \tfrac{m_{out}}{m_{in}}\overline{J}_E) \\
v_{r,11} &= \mu_r + h + \epsilon_r\gamma_T\tfrac{\overline{L}_{sD}}{\overline{F}_D} & v_{r,22} &= m_{out} + \mu_r + \mu_j + \alpha + \alpha\tfrac{\overline{L}_{sE}}{\overline{J}_E} \\
v_{r,32} &= -m_{out} & v_{r,33} &= m_{in} + \mu_r + \delta + \delta\overline{L}_{sU}/(\overline{F}_U + \tfrac{m_{out}}{m_{in}}\overline{J}_E).
\end{aligned} \qquad \text{(A28)}
$$

Seeking a threshold condition for invasion by resistant lice, we set $R_{0,r} > 1$ in equation (A27) and rearrange:

$$
\begin{aligned}
1 &< \frac{f_{r,11}}{v_{r,11}} + \frac{f_{r,33}}{v_{r,33}} - \frac{f_{r,11}}{v_{r,11}}\frac{f_{r,33}}{v_{r,33}} - \frac{f_{r,21}}{v_{r,11}}\frac{v_{r,32}}{v_{r,22}}\frac{f_{r,13}}{v_{r,33}} \\
\iff & \left(\frac{f_{r,11}}{v_{r,11}} - 1\right)\left(\frac{f_{r,33}}{v_{r,33}} - 1\right) < -\frac{f_{r,21}v_{r,32}f_{r,13}}{v_{r,11}v_{r,22}v_{r,33}} \\
\iff & (f_{r,11} - v_{r,11})(f_{r,33} - v_{r,33}) < -\frac{f_{r,21}v_{r,32}f_{r,13}}{v_{r,22}}.
\end{aligned}
\tag{A29}
$$

Substituting values from (A28) into (A29),

$$
\begin{aligned}
&R_{0,r} > 1 \iff \\
&\Big(\tfrac{\lambda_r}{c}\beta_{DD}\overline{F}_D - \mu_r - h - \epsilon_r\gamma_T\tfrac{\overline{L}_{sD}}{\overline{F}_D}\Big)\Big(\tfrac{\lambda_r}{c}\beta_{UU}\overline{F}_W - m_{in} - \mu_r - \delta - \delta\tfrac{\overline{L}_{sU}}{\overline{F}_W}\Big) \\
&\qquad\qquad < \frac{\tfrac{\lambda_r}{c}\beta_{ED}\overline{J}_E \cdot m_{out} \cdot \tfrac{\lambda_r}{c}\beta_{DE}\overline{F}_D\tfrac{m_{out}}{\sigma}\tfrac{\overline{J}_E}{\overline{F}_W}}{m_{out} + \mu_r + \mu_j + \alpha + \alpha\tfrac{\overline{L}_{sE}}{\overline{J}_E}},
\end{aligned}
\tag{A30}
$$

where $\overline{F}_W = \left(\overline{F}_U + \frac{m_{out}}{m_{in}}\overline{J}_E\right)$.

A.5 Population genetic approximation

We consider a sketch of a model in which resistance is determined by a diploid individual's genotype at a single resistance locus, with possible resistant and susceptible alleles. We assume that the frequency of the resistant allele is low, such that we can ignore homozygous resistant individuals (a common assumption in population genetic models). This leaves homozygous susceptible lice, with abundance L_{ss}, and heterozygous resistant lice, with abundance L_{rs}, present at frequencies $L_{ss}/(L_{ss} + L_{rs})$ and $L_{rs}/(L_{ss} + L_{rs})$, respectively. If we assume that lice mate randomly, then the rate of homozygous susceptible larval production, analogous to the first term in Equation 1, becomes:

$$
\underbrace{\lambda_s L_{ss}\frac{L_{ss}}{(L_{ss}+L_{rs})}}_{\substack{\text{production by susceptible lice}\\\text{mating with susceptible lice}}} + \underbrace{\tfrac{\lambda_s}{2}L_{ss}\frac{L_{rs}}{(L_{ss}+L_{rs})}}_{\substack{\text{production by susceptible lice}\\\text{mating with resistant lice}}} + \underbrace{\tfrac{\lambda_r}{2}L_{rs}\frac{L_{ss}}{(L_{ss}+L_{rs})}}_{\substack{\text{production by resistant lice}\\\text{mating with susceptible lice}}} + \underbrace{\tfrac{\lambda_r}{4}L_{rs}\frac{L_{rs}}{(L_{ss}+L_{rs})}}_{\substack{\text{production by resistant lice}\\\text{mating with resistant lice}}} .
\tag{A31}
$$

This simplifies to:

$$\lambda_s \frac{L_{ss}^2}{(L_{ss} + L_{rs})} + \tfrac{(\lambda_s+\lambda_r)}{2} \frac{L_{ss}L_{rs}}{(L_{ss} + L_{rs})} + \tfrac{\lambda_r}{4} \frac{L_{rs}^2}{(L_{ss} + L_{rs})}. \tag{A32}$$

By similar logic, the rate of resistant louse production is:

$$\underbrace{\tfrac{\lambda_s}{2} L_{ss} \frac{L_{rs}}{(L_{ss} + L_{rs})}}_{\substack{\text{production by susceptible lice} \\ \text{mating with resistant lice}}} + \underbrace{\tfrac{\lambda_r}{2} L_{rs} \frac{L_{ss}}{(L_{ss} + L_{rs})}}_{\substack{\text{production by resistant lice} \\ \text{mating with susceptible lice}}} + \underbrace{\tfrac{\lambda_r}{2} L_{rs} \frac{L_{rs}}{(L_{ss} + L_{rs})}}_{\substack{\text{production by resistant lice} \\ \text{mating with resistant lice}}}, \tag{A33}$$

which simplifies to:

$$\tfrac{(\lambda_s+\lambda_r)}{2} \frac{L_{ss}L_{rs}}{(L_{ss} + L_{rs})} + \tfrac{\lambda_r}{2} \frac{L_{rs}^2}{(L_{ss} + L_{rs})}. \tag{A34}$$

Inserting Expressions A32 and A34 as the louse-production terms in our full model (Equations 4), and leaving mortality and migration rates unaffected, we get a model that can describe the spread of resistance at low frequency. While the dynamics of the full model differ from our main, simpler case, the R_0 calculations for the invasion of resistance are almost identical.

Linearising the new model's generic resistant-louse production term about the resistance-free equilibrium, we get:

$$\frac{d}{dL_{rs}} \left(\tfrac{(\lambda_s+\lambda_r)}{2} \frac{L_{ss}L_{rs}}{(L_{ss} + L_{rs})} + \tfrac{\lambda_r}{2} \frac{L_{rs}^2}{(L_{ss} + L_{rs})} \right) \Bigg|_{L_{rs}=0, L_{ss}=\bar{L}_{ss}} = \frac{(\lambda_s + \lambda_r)}{2}. \tag{A35}$$

Thus, the relevant **F** and **V** matrices for the population-genetic model are identical to those of our main model, except that λ_r in Equations A28 is replaced by $(\lambda_s + \lambda_r)/2$. If we retain the simplifying assumption (used throughout) that susceptible and resistant lice produce larvae at the same rate, the R_0 value for invasion of heterozygous resistance is identical to that in Equations A27 and A28.

A.6 Supplemental results

The following results present simulated equilibrium conditions, across a range of parameter combinations, following inoculation with a single resistant louse. In each case we calculated the net reproductive number for resistant lice in the system, $R_{0,r}$, at the time of inoculation, and at the

final equilibrium we recorded the proportion of farm lice that were resistant, $L_{rD}/(L_{rD}+L_{sD})$. We also considered the total number of lice per farm host, $(L_{rD} + L_{sD})/F_D$; the size of the exposed wild population, J_E; and the growth rate of resistant lice on farms, dL_r/dt, calculated a short time (five time steps) after inoculation to allow the behaviour of the system to adjust after perturbation (Appendix A.6).

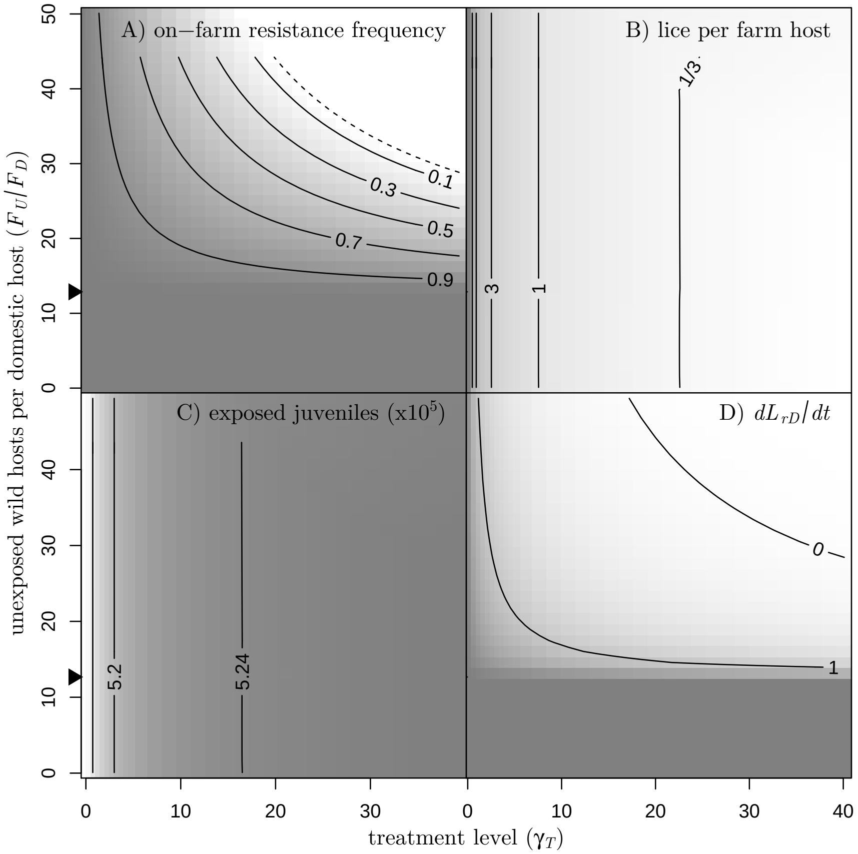

Figure A1: Effects on the emergence of treatment resistance in sea lice on salmon farms of the rate of treatment increase per louse per domestic host (γ_T) and the size of the unexposed wild host population, relative to the domestic host population, (F_U/F_D). Shown are A) the proportion of domestic lice that were resistant, B) the number of lice on each domestic host, and C) the number of exposed juveniles at equilibrium, and D) the annual rate of increase in resistant lice soon after inoculation with a single resistant louse. The dashed line in A) shows the threshold value of the net reproductive number for resistant lice, $R_{0,r}$=1, below which resistance cannot spread. ▶ indicates the threshold unexposed population size required to sustain lice in the system, in the absence of farms (see Equation A20). A) through D) are results from a differential-equation model tracking treatment-resistant and treatment-susceptible sea-louse parasites, carried between domestic and unexposed wild populations by an exposed wild host population. See text for details.

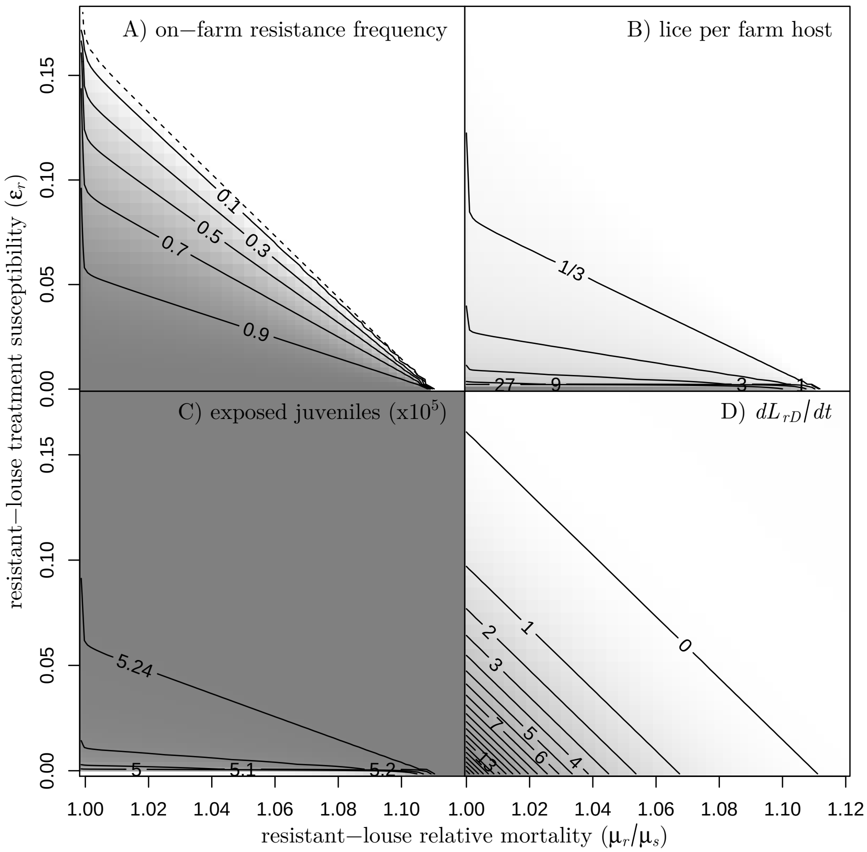

Figure A2: Effects relevant to the emergence of treatment resistance in sea lice on salmon farms of resistance costs, measured as mortality rate relative to that for susceptible lice (μ_r/μ_s), and resistant-louse treatment susceptibility (ϵ_r). Shown are A) the proportion of domestic lice that were resistant, B) the number of lice on each domestic host, and C) the number of exposed juveniles at equilibrium, and D) the annual rate of increase in resistant lice soon after inoculation with a single resistant louse. The dashed line in A) shows the threshold value of the net reproductive number for resistant lice, $R_{0,r}$=1, below which resistance cannot spread. A) through D) are results from a differential-equation model tracking treatment-resistant and treatment-susceptible sea-louse parasites, carried between domestic and unexposed wild populations by an exposed wild host population. See text for details

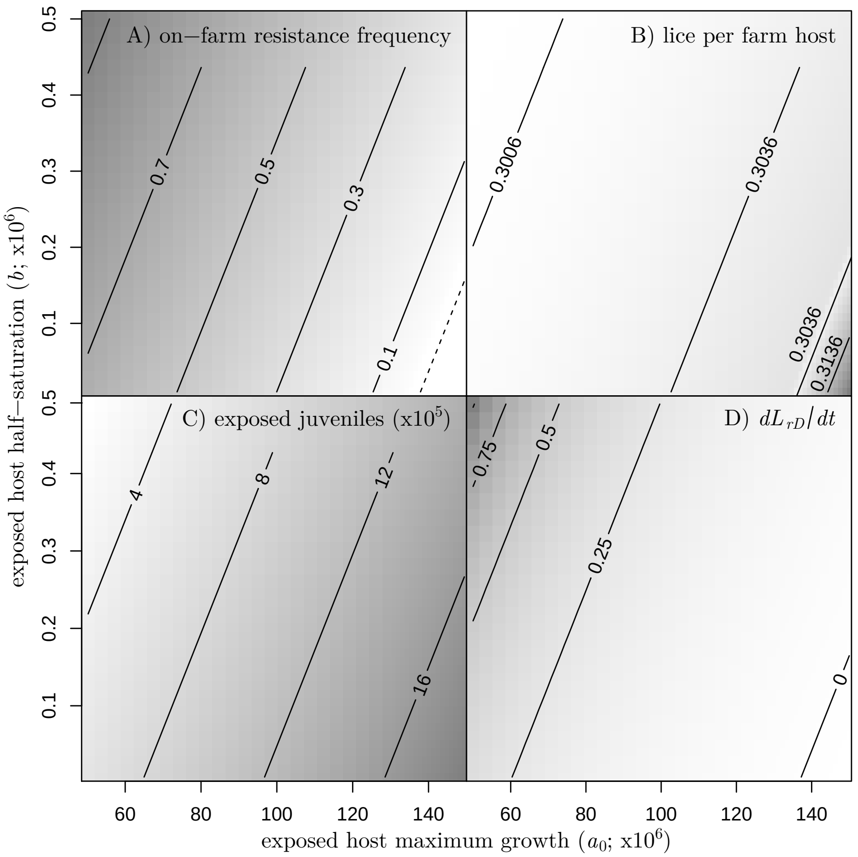

Figure A3: Effects on the emergence of treatment resistance in sea lice on salmon farms of the maximum (a_0) and half-saturation level (b) for the growth rate of exposed wild-salmon hosts. Shown are A) the proportion of domestic lice that were resistant, B) the number of lice on each domestic host, and C) the number of exposed juveniles at equilibrium, and D) the annual rate of increase in resistant lice soon after inoculation with a single resistant louse. The dashed line in A) shows the threshold value of the net reproductive number for resistant lice, $R_{0,r}$=1, below which resistance cannot spread. A) through D) are results from a differential-equation model tracking treatment-resistant and treatment-susceptible sea-louse parasites, carried between domestic and unexposed wild populations by an exposed wild host population. See text for details

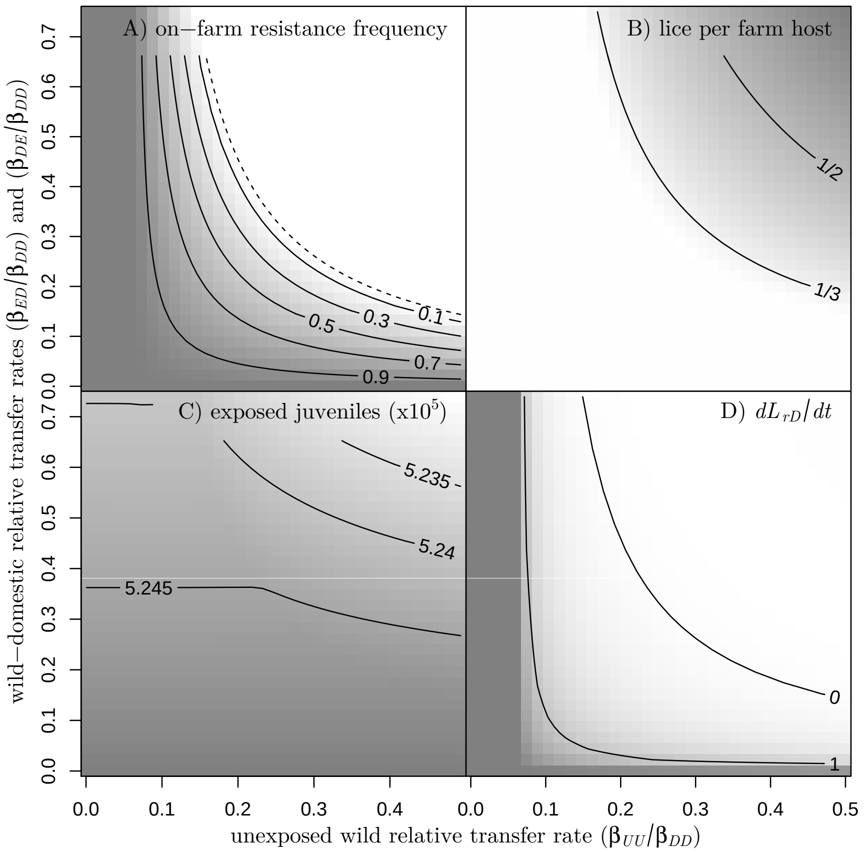

Figure A4: Effects relevant to the emergence of treatment resistance in sea lice on salmon farms of louse transmission rates between exposed and domestic hosts (β_{ED} and β_{DE}) and within the unexposed population (β_{UU}), relative to transmission among domestic hosts (β_{DD}). Shown are A) the proportion of domestic lice that were resistant, B) the number of lice on each domestic host, and C) the number of exposed juveniles at equilibrium, and D) the annual rate of increase in resistant lice soon after inoculation with a single resistant louse. The dashed line in A) shows the threshold value of the net reproductive number for resistant lice, $R_{0,r}$=1, below which resistance cannot spread. A) through D) are results from a differential-equation model tracking treatment-resistant and treatment-susceptible sea-louse parasites, carried between domestic and unexposed wild populations by an exposed wild host population. See text for details
